# Hamartoma-like lesions in the mouse retina: an animal model of *Pten* hamartoma tumour syndrome

**DOI:** 10.1242/dmm.031005

**Published:** 2018-05-21

**Authors:** Nobuhiko Tachibana, Yacine Touahri, Rajiv Dixit, Luke Ajay David, Lata Adnani, Robert Cantrup, Tooka Aavani, Rachel O. Wong, Cairine Logan, Kyle C. Kurek, Carol Schuurmans

**Affiliations:** 1 Biological Sciences Platform, Sunnybrook Research Institute, Room 116, 2075 Bayview Ave, Toronto, ON, M4N 3M5, Canada; 2 Department of Biochemistry and Molecular Biology, Alberta Children's Hospital Research Institute, Hotchkiss Brain Institute, University of Calgary, Calgary, AB, T2N 4N1, Canada; 3 Department of Biological Structure, University of Washington, Seattle, WA 98195-7420, USA; 4 Department of Cell Biology and Anatomy, University of Calgary, Calgary, AB, T2N 4N1, Canada; 5 Department of Pathology and Laboratory Medicine, Alberta Children's Hospital Research Institute, University of Calgary, Calgary, AB, T2N 4N1, Canada

**Keywords:** PHTS, Hamartoma, Retinal malformation, Pten phosphatase, Non-cell autonomy, Drug therapy

## Abstract

*PTEN* hamartoma tumour syndrome (PHTS) is a heterogeneous group of rare, autosomal dominant disorders associated with *PTEN* germline mutations. PHTS patients routinely develop hamartomas, which are benign tissue overgrowths comprised of disorganized ‘normal’ cells. Efforts to generate PHTS animal models have been largely unsuccessful due to the early lethality of homozygous germline mutations in *Pten*, together with the lack of hamartoma formation in most conditional mutants generated to date. We report herein a novel PHTS mouse model that reproducibly forms hamartoma-like lesions in the central retina by postnatal day 21. Specifically, we generated a *Pten* conditional knockout (cKO) using a retinal-specific *Pax6::Cre* driver that leads to a nearly complete deletion of *Pten* in the peripheral retina but produces a mosaic of ‘wild-type’ and *Pten* cKO cells centrally. Structural defects were only observed in the mosaic central retina, including in Müller glia and in the outer and inner limiting membranes, suggesting that defective mechanical integrity partly underlies the hamartoma-like pathology. Finally, we used this newly developed model to test whether rapamycin, an mTOR inhibitor that is currently the only PHTS therapy, can block hamartoma growth. When administered in the early postnatal period, prior to hamartoma formation, rapamycin reduces hamartoma size, but also induces new morphological abnormalities in the *Pten* cKO retinal periphery. In contrast, administration of rapamycin after hamartoma initiation fails to reduce lesion size. We have thus generated and used an animal model of retinal PHTS to show that, although current therapies can reduce hamartoma formation, they might also induce new retinal dysmorphologies.

This article has an associated First Person interview with the first author of the paper.

## Abstract (precis)

**Summary:** The authors present the first PHTS animal model that successfully recapitulates hamartoma formation in the retina and can be used to assess drug therapies.

## INTRODUCTION

The basic body plan is established during development, dictating the size and positioning of each cell type for optimal tissue functioning. *PTEN* (phosphatase and tensin homolog) is a well-known negative regulator of cell growth and an essential determinant of tissue patterning (Cantrup et al., 2012; Yamada and Araki, 2001). It encodes a lipid and protein phosphatase that controls the phosphorylation status of membrane phospholipids by removing a 3′-phosphate from PIP3 [phosphatidylinositol-(3,4,5)-trisphosphate] to convert it to PIP2 [phosphatidylinositol-(4,5)-bisphosphate], thus counteracting the activity of phosphoinositide-3-kinase (PI3K), which phosphorylates PIP2 to generate PIP3. The conversion of PIP3 to PIP2 alters downstream signalling as PIP3 is a second messenger that controls multiple cellular processes, including polarity, proliferation, survival, growth and migration (Comer and Parent, 2007; Stambolic et al., 1998). Mutation of *PTEN* results in elevated signalling downstream of PIP3, including activation of the mTOR pathway, a major regulator of cell growth and a target of rapamycin.

In humans, various autosomal dominant germline mutations in *PTEN*, ranging from missense point mutations to frameshift deletion mutations, are associated with *PTEN* hamartoma tumour syndrome (PHTS), a heterogeneous spectrum of disorders ranging from autism spectrum disorder (ASD) and brain patterning defects (Lhermitte–Duclos disease) to cancer predisposition syndromes (Cowden syndrome) (Hollander et al., 2011; Kurek et al., 2012a; Pilarski et al., 2011). A unifying feature of PHTS is the formation of multiple congenital malformations known as hamartomas, which are benign tissue overgrowths consisting of disordered ‘normal’ cellular elements. Despite phenotypic variability, all PHTS patients develop hamartomas, and these lesions can arise in all embryological lineages, but are most common in the skin, connective tissue, vasculature, gastrointestinal tract and central nervous system (CNS), including the retina (Echevarria et al., 2014; Mansoor and Steel, 2012; Pilarski et al., 2013). Among the most common are debilitating soft tissue lesions that cause significant morbidity and mortality. Formation of CNS hamartomas can also have devastating consequences, resulting in neurological dysfunction such as epilepsy, ASD and vision loss (Echevarria et al., 2014; Mansoor and Steel, 2012; Pilarski et al., 2013).

The dysregulation of postnatal tissue growth associated with PHTS not only results in hyperplasia, but also in an increased risk of malignant transformation, especially in the breast, thyroid and endometrium. Thrombosis and cardiac failure are also known complications (Kurek et al., 2012b). Surgical treatments are challenging, especially with such a multifocal disease. Isolated case reports document some benefit from non-invasive drug treatments targeting PI3K-AKT-mTOR pathway inhibition using sirolimus (also known as rapamycin), but efficacy plateaus after several months and is not durable following cessation (Iacobas et al., 2011; Marsh et al., 2008). Additional benefits have been documented *in vitro* using a combination of targeted therapies to components of the PTEN pathway (Schmid et al., 2014; Wang et al., 2007). However, it is unclear how long-term suppression of this vital pathway will affect growth and development during childhood and adolescence, presumably the optimal window for treatment. Nevertheless, because PHTS hamartomas are comprised of non-transformed cells, they may be highly amenable to correction using novel therapies targeting cell growth and patterning that may also prevent subsequent malignant transformation.

The design of novel therapies for PHTS would be greatly facilitated by animal models, but currently there are very few models of PHTS, especially in the CNS, highlighting the difficulty in replicating this disease. One reason may be that hamartomas form in tissues where there is a mosaic of *Pten* mutant and wild-type cells. In support of this notion, hamartomas associated with mutations in *TSC1* or *TSC2* (tuberous sclerosis complex 1 and 2) genes in humans (van Eeghen et al., 2012) have been phenocopied in zebrafish by the generation of mosaic embryos that carry wild-type and *tsc2* (vu242/vu242) mutant cells (Kim et al., 2011). Here, we created a unique mouse model that recapitulates the PHTS disease process associated with human *PTEN* mutations, demonstrating that the conditional knockout (cKO) of *Pten* in a mosaic fashion in the central retina, resulting in a mix of wild-type and mutant cells, leads to hamartoma formation. Using this model, we subsequently tested the *in vivo* efficacy of sirolimus (rapamycin), a current drug therapy, which we found reduces hamartoma size but also significantly disrupts the morphology of the peripheral retina, where *Pten* is uniformly deleted. Notably, these effects of sirolimus were only observed when it was administered in the early postnatal period; treatment post-hamartoma development had no effect on hamartoma size or retinal morphology. Thus, our creation of a new PHTS animal model opens the door for the testing of new therapeutic agents and, while the adverse effects of sirolimus make it a poor choice for the treatment of retinal hamartomas, other more effective drug therapies may be uncovered using this model system.

## RESULTS

### Hamartoma-like lesions develop in the central region of *Pten* cKO retinas in the early postnatal period

**Fig. 1.**
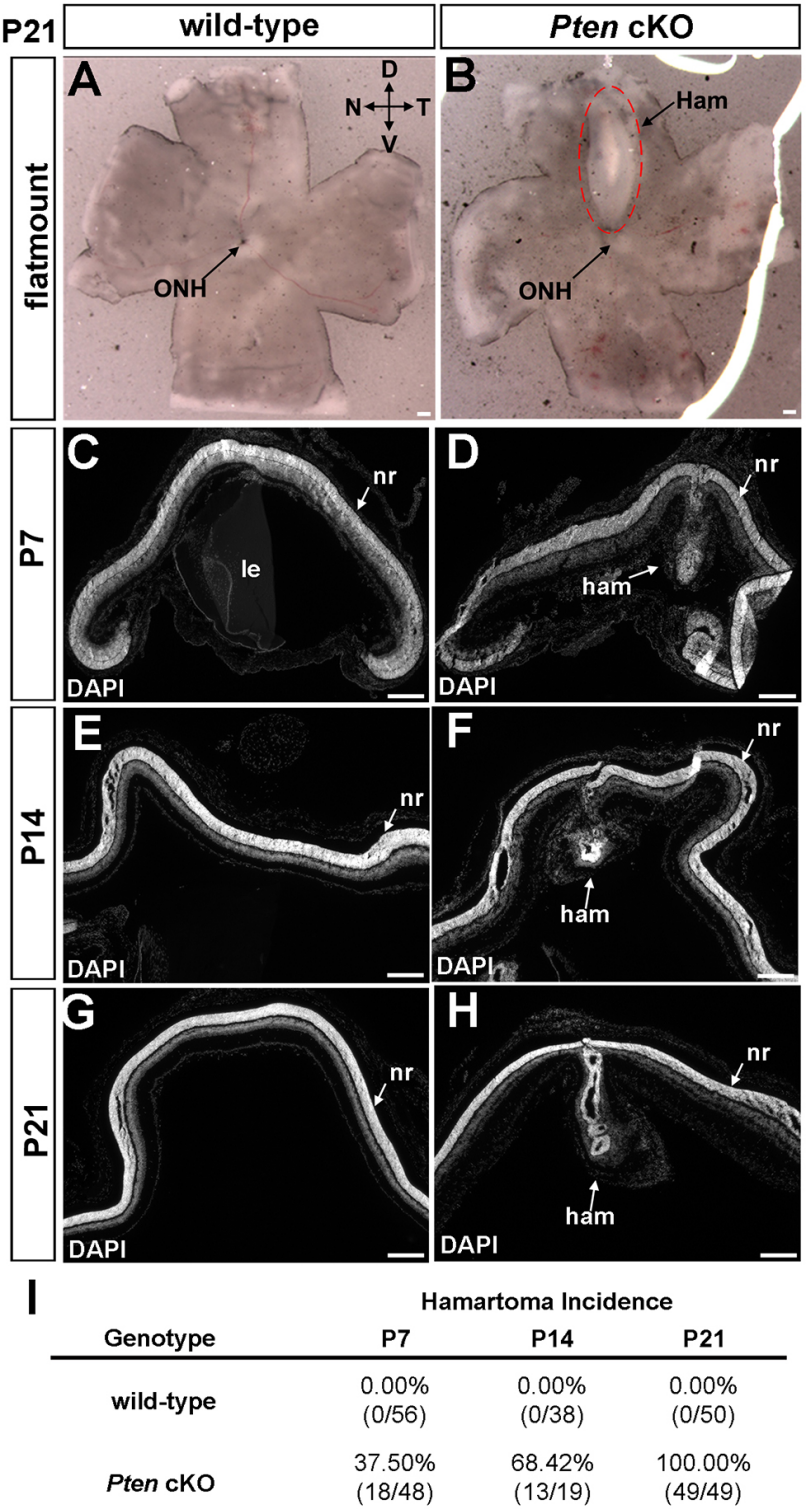
**Hamartoma-like lesions form in the dorsal-central retina in *Pten* cKOs in the early postnatal period.** (A,B) Flat-mount images of P21 wild-type (A) and *Pten* cKO (B) retinas. The dorsal-central retina is dysmorphic in *Pten* cKOs (red dashed line). (C-H) DAPI staining of transverse sections through the retinas of wild-type and *Pten* cKOs at P7 (C,D), P14 (E,F) and P21 (G,H). (I) Incidence of hamartoma-like lesions at P7, P14 and P21. (P7 wild-type: *n*=56, P7 *Pten* cKO: *n*=48, P14 wild-type: *n*=38, P14 *Pten* cKO: *n*=19, P21 wild-type: *n*=50, P21 *Pten* cKO: *n*=49.) D, dorsal; Ham, hamartoma; le, lens; N, nasal; nr, neural retina; ONH, optic nerve head; T, temporal; V, ventral. Scale bars: 200 μm.

We previously reported the generation of a *Pten* cKO using a *Pax6::Cre* driver that is more active in the peripheral versus central retina (Cantrup et al., 2012; Marquardt et al., 2001; Tachibana et al., 2016). Strikingly, we noted that, in postnatal day (P)21 *Pten* cKO mice (*Pten^fl/fl^;Pax6::Cre^+^*), a bulge-like ectopic mass developed in a site-specific manner on the dorsal-medial retinal surface in a completely penetrant manner (*n*=49/49; Fig. 1B). These lesions resembled the hamartomas characteristic of PHTS, and hereafter are referred to as hamartoma-like, whereas no such masses were detected in P21 wild-type animals (Fig. 1A) or in animals heterozygous for a *Pten* mutation (*Pten^fl/+^;Pax6::Cre^+^*; data not shown).

We questioned whether the *Pten* cKO central lesions were developmental defects, or whether they arose post-cellular differentiation, which is complete by P7 in the central retina and P12 in the peripheral retina (Young, 1985). By examining retinas between P7, the earliest time point we detected the hamartoma-like lesions, and P21, we revealed that central morphological defects developed progressively over time, with 37.5% (*n*=18/48), 68.42% (*n*=13/19) and 100% (*n*=49/49) of *Pten* cKO retinas displaying central dysmorphologies that were not detectable in wild-type retinas at P7 (Fig. 1C,D), P14 (Fig. 1E,F) and P21 (Fig. 1G,H), respectively (Fig. 1I). The central retinal lesions in *Pten* cKOs thus begin to form during the late stages of cellular differentiation and become completely penetrant soon thereafter.

### Hamartoma-like lesions in *Pten* cKOs consist of a mosaic of recombined mutant cells and ‘wild-type’ cells that retain Pten expression

**Fig. 2.**
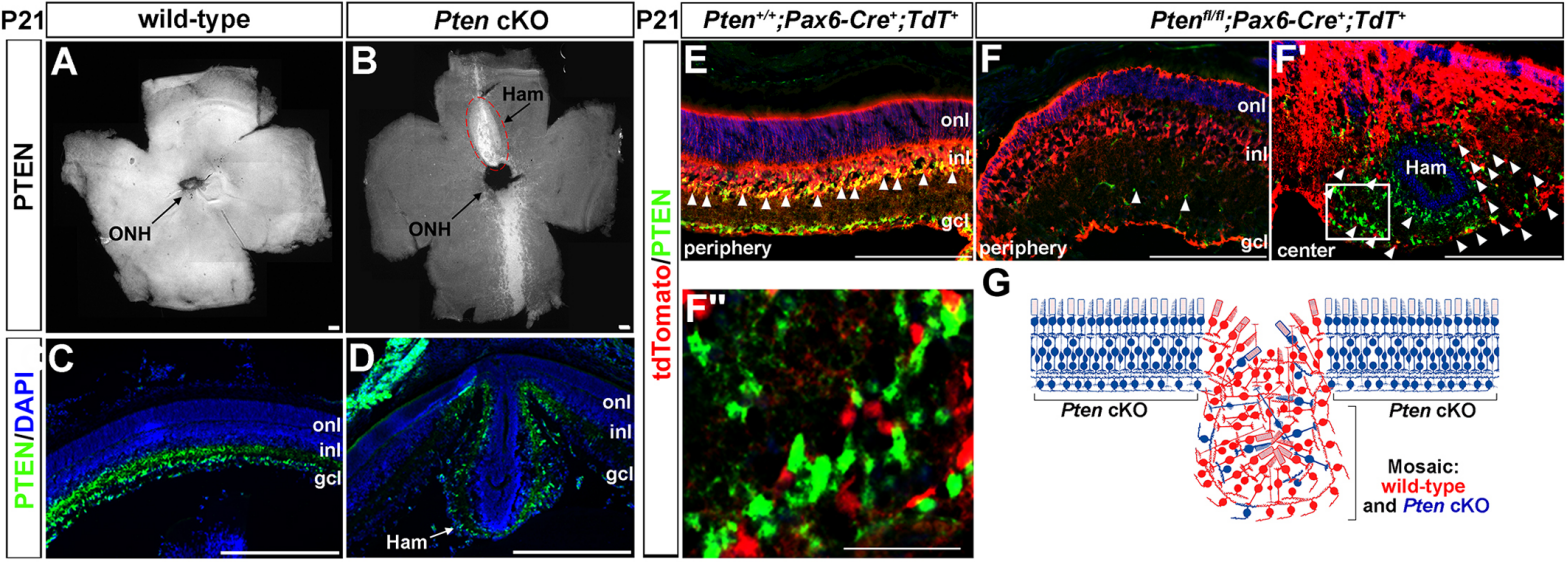
**The central hamartoma-like lesions contain a mix of *Pten*-expressing ‘wild-type’ cells and *Pten* mutant cells.** (A-D) Pten immunostaining of flat-mount (A,B) and transverse (C,D) sections of P21 wild-type (A,C) and *Pten* cKO (B,D) retinas. Blue is DAPI counterstain in C,D. Pten expression is retained in the central retina in *Pten* cKOs, including in the site where the dorsal lesion forms (red hashed line), indicating that the *Pax6::Cre* driver is less active in this domain. (E-F″) Pten (green) and tdTomato (red) expression in transverse sections of a P21 *Pten^+/+^;Pax6::Cre^+^;Rosa-tdTomato^+^* (wild-type; E) retina and P21 *Pten^fl/fl^;Pax6::Cre^+^;Rosa-tdTomato^+^* (*Pten* cKO) retinas in the peripheral (F) and central (F′,F″) retina. Arrowheads in E mark tdTomato^+^/Pten^+^ cells in which the Cre driver is active. Arrowheads in F mark the few Pten^+^ cells, which are not tdTomato^+^, which have retained Pten expression because the Cre driver was not active. Arrowheads in F′ mark the tdTomato^+^ cells (red) that have undergone recombination and are therefore *Pten* mutant cells, which are interspersed between Pten^+^ ‘wild-type’ cells (green). F″ is a magnified view of the boxed area in F′. (G) Schematic illustration of the central lesion in *Pten* cKOs, showing the mix of ‘wild-type’ (red) and *Pten* mutant (blue) cells in the central hamartoma. gcl, ganglion cell layer; Ham, hamartoma; inl, inner nuclear layer; ONH, optic nerve head; onl, outer nuclear layer; *tdT; Rosa-tdTomato*. Scale bars: 200 μm.

The spatial specificity of the hamartoma-like lesions coincided with the previously reported dorsal-central gap in *Pax6::Cre* driver activity (Marquardt et al., 2001). We confirmed this finding, revealing that, although there was wide-spread Pten expression in flat-mounts of wild-type retinas (Fig. 2A), Pten expression was only retained in a medial stripe in *Pten* cKO retinas (Fig. 2B). Notably, even though Pten expression was also maintained in a thin ventral stripe in the central retina (Fig. 2B), retinal hamartomas were only ever observed in dorsal regions, which is the region that we focused on for the remainder of our study.

To better assess Pten expression in the hamartoma-like lesions, we sectioned P21 retinas, revealing that Pten was normally expressed throughout the lower inner nuclear layer (INL) and ganglion cell layer (GCL) in wild-type retinas (Fig. 2C), whereas expression was only detected in scattered cells in the central dysmorphic region of *Pten* cKOs (Fig. 2D). Given that Pten was not expressed in all retinal cells (this study and Cantrup et al., 2012), it was not clear whether the non-expressing cells within the central *Pten* cKO lesions also included some *Pten* ‘wild-type’ (i.e. non-recombined) cells. To address this issue, we performed lineage tracing, introducing a *Rosa-tdTomato* transgenic reporter into animals heterozygous (*Pten^fl/+^;Pax6::Cre^+^*; Fig. 2E) or homozygous (*Pten^fl/fl^;Pax6::Cre^+^*; Fig. 2F,F″) for a *Pten* mutation. In P21 *Pten* heterozygotes, tdTomato^+^ cells were detected throughout the retinal layers, which was expected given that Pten is expressed at earlier stages in most retinal progenitor cells, which then give rise to neurons in each of the retinal layers (Cantrup et al., 2012; Marquardt et al., 2001). Notably, the highest tdTomato expression was observed in the INL and GCL, coinciding with the Pax6 expression domain at P21 (i.e. the site of the highest Cre activity; Fig. 2E). In the periphery of *Pten* cKO retinas, tdTomato was similarly expressed in all retinal layers, with the highest levels observed in the disorganized INL and GCL. In contrast, virtually no Pten expression was detected in the peripheral retina, except in a very small number of cells, suggesting that Cre recombination was nearly complete in this domain (Fig. 2F). Conversely, in the central retina of *Pten* cKOs, whereas Pten expression was retained in a large number of cells, there were also some tdTomato^+^ cells present, indicating that the central hamartoma-like lesions comprise a mosaic of *Pten* mutant and *Pten* ‘wild-type’ cells (Fig. 2F-F″,G).

### *Pten* cKO retinal lesions resemble hamartomas and comprise normal retinal cell types

The retina is an ideal tissue to follow hamartoma formation as it has a very precise epithelial structure and is made up of defined cell types, allowing disease progression to be easily visualized. The retina is normally subdivided into three cellular layers: (1) the outer nuclear layer (ONL), which contains rod and cone photoreceptors that respond to light and transmit impulses to (2) the INL, where three interneuron populations (amacrine, bipolar and horizontal cells) modulate and process signals that are transmitted to retinal ganglion cells (RGCs), the output neurons in (3) the GCL that send axon tracts to the brain. Also located in the INL are Müller glia, the predominant glial cell type. The normal tri-laminated structure of the retina was obviously perturbed in the central hamartomas of *Pten* cKO retinas (Fig. 1C versus D, E versus F, G versus H). Given that one of the hallmark features of hamartomas in other tissues is the presence of a normal complement of non-transformed cells, we asked whether each of the seven retinal cell types were present in the *Pten* cKO retinal hamartomas.

**Fig. 3.**
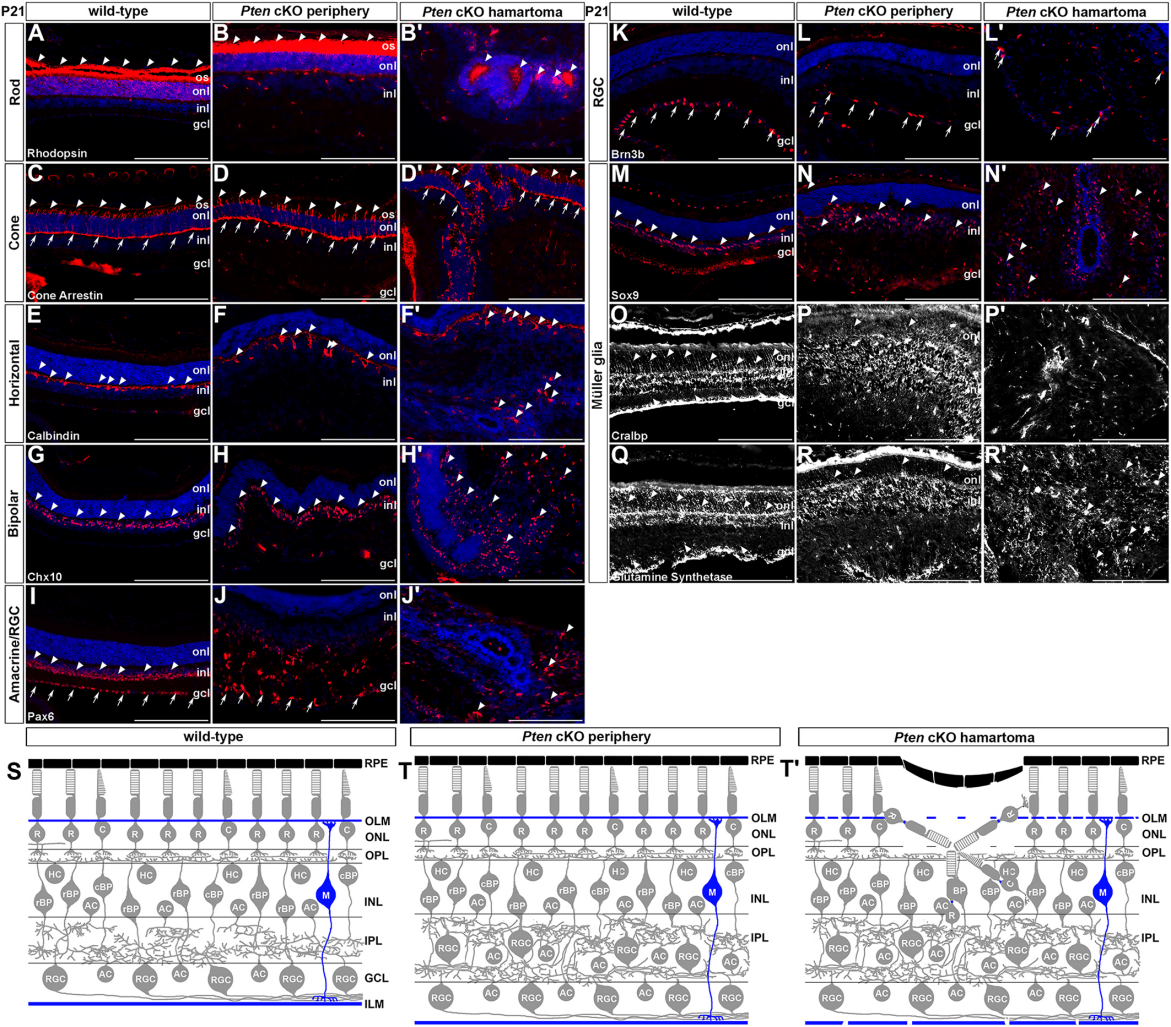
**All seven retinal cell types are present in *Pten* cKO hamartomas.** (A-R′) Immunolabelling of P21 wild-type and *Pten* cKO retinas for rhodopsin (A-B′; marks rods), cone arrestin (C-D′; marks cones), calbindin (E-F′; marks horizontal cells), Chx10 (G-H′; marks bipolar cells), Pax6 (I-J′; marks amacrine cells and RGCs), Brn3b (K-L′; marks RGCs), Sox9 (M-N′; marks Müller glia), Cralbp (O-P′; marks Müller glia) and glutamine synthetase (Q-R′; marks Müller glia). Blue is DAPI counterstain. Arrowheads indicate cells that are positive for the markers. Arrows in C-D' mark cone pedicles. Arrows in I-L' mark labeled cells in the GCL. gcl, ganglion cell layer; inl, inner nuclear layer; ILM, inner limiting membrane; IPL, inner plexiform layer; OPL, outer plexiform layer; OLM, outer limiting membrane; onl, outer nuclear layer; os, outer segment; RPE, retinal pigment epithelium. Scale bars: 200 μm.

To test for the presence of rod photoreceptors, we examined the expression of rhodopsin, which is expressed at low levels in rod cell bodies and at high levels in the outer segments in wild-type P21 retinas (Fig. 3A). Rhodopsin expression was also observed in rod cell bodies and in the outer segments in the P21 *Pten* cKO peripheral retina, where *Pten* was deleted (Fig. 3B). In contrast, in the central dysmorphic region of *Pten* cKO retinas, rhodopsin was expressed in the central lumen of the rosette-like hamartoma lesions, indicative of an invagination event such that the rod outer segments were no longer pointing outwards (Fig. 3B′). Consistent with this interpretation, a similar invagination was seen when examining the expression of cone arrestin, which is normally expressed in the outer segments in wild-type (Fig. 3C) and *Pten* cKO (Fig. 3D) peripheral cone photoreceptors. In contrast, cone arrestin staining was observed in the internal lumen in the central lesions observed in *Pten* cKOs (Fig. 3D′). The misorientation of cone photoreceptors was observed for both M- and S-cones as revealed by the expression of M-opsin (Fig. S1A-B′) and S-opsin (Fig. S1C-D′), respectively, which were both detected in the central lumens of the rosette-like structures in P21 *Pten* cKO retinas. The outer segments are thus oriented towards the central lumens of the hamartoma-like lesions, rather than in their normal configuration pointing towards the apical side of the retinal tissue.

We next examined the interneuron populations that make up the INL. In P21 wild-type retinas, calbindin^+^ horizontal cells formed a highly dispersed monolayer in the upper INL (Fig. 3E), Chx10^+^ bipolar cells were organized in a much more dense cell layer in the upper INL (Fig. 3G), and Pax6^+^ amacrine cells were densely packed in the lower INL (Fig. 3I). Notably, Pax6 also marked displaced amacrine cells and RGCs in the GCL of P21 wild-type retinas (Fig. 3I). In contrast, in the periphery of P21 *Pten* cKO retinas, where *Pten* is deleted, all three interneuron populations were present, but calbindin^+^ horizontal cells (Fig. 3F), Chx10^+^ bipolar cells (Fig. 3H) and Pax6^+^ amacrine cells (Fig. 3J) did not form tight layers but were instead dispersed throughout the expanded INL and in the normally cell-sparse inner plexiform layer (IPL), as previously reported (Cantrup et al., 2012). In the central hamartoma tissue, all three interneuron populations were also present, but there was no laminar organization and cells marked with calbindin (Fig. 3F′), Chx10 (Fig. 3H′) and Pax6 (Fig. 3J′) were aberrantly distributed throughout the tissue. Similarly, although Brn3b marked RGCs in the GCL in P21 wild-type retinas (Fig. 3K), it marked a less distinct cell layer in the peripheral P21 *Pten* cKO retinal tissue (Fig. 3L) and was even more disorganized in the *Pten* cKO central lesion (Fig. 3L′). Finally, Müller glia cell bodies, which align in the upper INL (Fig. 3M), were disorganized in *Pten* mutant peripheral tissue (Fig. 3N), especially in the central hamartoma lesion (Fig. 3N′). Thus, all seven retinal subtypes were indeed present in the central hamartoma-like mass in *Pten* cKO retinas, albeit disorganized, suggesting that these lesions were indeed hamartomas, consisting of normal differentiated retinal cells (Fig. 3S-T′).

### Only a few ectopic dividing cells are observed in the *Pten* cKO hamartoma-like lesions

*PTEN* germline mutations are associated not only with PHTS, which is a benign disease, but also with malignant transformation, with somatic *PTEN* mutations being acquired in many aggressive cancers. One of the features of transformed tissue is the re-entry of post-mitotic differentiated cells into the cell cycle. We thus questioned whether the central hamartoma-like lesions observed in *Pten* cKO retinas were hamartomas or whether they resembled tumours, showing proliferation as a sign of malignancy.

We first assessed cell division in P7 retinas, at the time when hamartoma formation was first observed. At P7, Ki67^+^ dividing retinal progenitors were mainly confined to the ciliary margins and peripheral neural retina in both wild-type (Fig. S2B,B′) and *Pten* cKO (Fig. S2D,D′) pups, and proliferation rates were equivalent in both genotypes (wild type: 15±1.52 Ki67^+^ cells; *Pten* cKO: 17.22±0.79, *P*=0.43; Fig. S2E). In contrast, no proliferating cells were detected in the central retina at P7 in wild-type animals (Fig. S2A,A′). Although there were a few Ki67^+^ cells in the hamartoma-like masses in *Pten* cKOs (Fig. S2C,C′), and these numbers were significantly higher than in wild-type animals (wild-type: 0 versus *Pten* cKO: 1.8±0.62, *P*<0.013), the number of dividing cells was very low (Fig. S2E), suggesting that proliferation was not a large contributor to mass formation. Indeed, by P21, there were virtually no Ki67^+^ dividing cells observed in the central or peripheral retina in either wild-type (Fig. S3A,A′,E,E′) or *Pten* cKO (Fig. S3C,C′,G,G′) animals, except in the very peripheral ciliary margins.

In summary, given that the abnormal masses found in the central retina of *Pten* cKOs have very few proliferating cells, and largely retain Pten expression, these malformations are unlikely to be malignant, and instead mimic a benign hamartoma.

### Disorganization of Müller glia and a loss of OLM integrity on the apical side of *Pten* cKO retinas

We next sought to determine how the central retina might become progressively disorganized in *Pten* cKOs by examining whether known structural elements were perturbed. Structural integrity is maintained on the apical side of the retina by the retinal pigment epithelium (RPE) and the outer limiting membrane (OLM), and on the basal side by the inner limiting membrane (ILM) (Galli-Resta et al., 2008). The OLM is formed by the apical endfeet of Müller glia, which form adherens junctions with photoreceptor cell inner segments, and the ILM is a basement membrane comprising extracellular matrix components (e.g. laminin, fibronectin) that are contacted by Müller glia endfeet (Galli-Resta et al., 2008). Müller glia thus have a central role in providing structural integrity and mechanical strength to the retina, which they achieve by sending processes that contact both the basal ILM and apical OLM.

To visualize Müller glia processes, we first immunostained P21 wild-type retinas with CRALBP (Fig. 3O) and glutamine synthetase (Fig. 3Q), revealing that these markers labelled fine processes that projected towards the basal surface of the retina on one side, and apically through the ONL towards the photoreceptor inner segments on the other side. In contrast, in P21 *Pten* cKOs, although CRALBP (Fig. 3P) and glutamine synthetase (Fig. 3R) immunostaining was observed in the peripheral retina, including in some correctly oriented processes, there was a complete loss of Müller glial cell organization in the central hamartoma-like lesions (Fig. 3P′,R′), even though these cells were present based on Sox9 staining (Fig. 3N′). These data suggested that the OLM and ILM, which comprise Müller glia processes, might also not develop normally in *Pten* cKO hamartomas, which we investigated further.

**Fig. 4.**
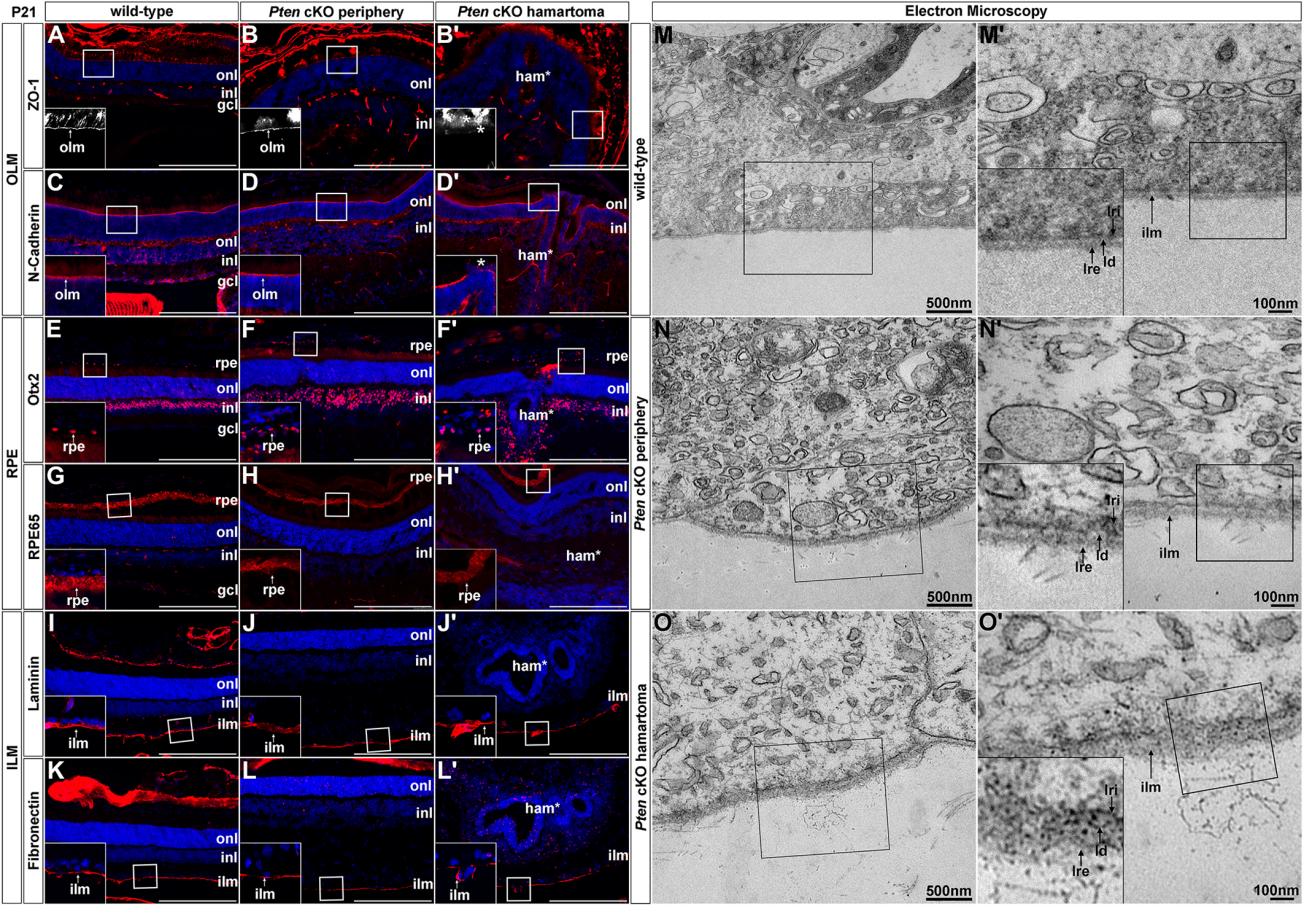
**Disruption of the OLM and to a lesser extent the ILM in *Pten* cKO retinas.** (A-L′) Immunolabelling of P21 wild-type and *Pten* cKO retinas for ZO-1 (A-B′), N-cadherin (C-D′), Otx2 (E-F′), RPE65 (G-H′), laminin (I-J′) and fibronectin (K-L′). Blue is DAPI counterstain. Insets in A-L′ are 2.5-fold magnifications of the boxed areas in the larger images. Loss of integrity of the OLM in the *Pten* cKO peripheral retina and hamartoma is indicated with asterisks in B′,D′. Scale bars: 200 μm. (M-O′) Electron microscopy of P21 wild-type (M,M′) and *Pten* cKO retinas in the periphery (N,N′) and hamartoma region (O,O′), focusing on the ILM. M′-O′ are 2.5-fold magnifications of the boxed areas in M-O, respectively. Insets in M′-O′ are 2-fold magnifications of boxed areas in M′-O′. gcl, ganglion cell layer; ham, hamartoma; ilm, inner limiting membrane; inl, inner nuclear layer; ld, lamina densa; lre, lamina rara externa; lri, lamina rara interna; NR, neural retina; onl, outer nuclear layer; rpe, retinal pigment epithelium.

To further study the integrity of the OLM in *Pten* cKOs, we labelled the OLM using zonula occludens-1 (ZO-1), which is a junctional protein that is required for the positioning of adherens junctions in the OLM (Pearson et al., 2010). ZO-1 labelled a continuous line of junctional complexes overlying the ONL in P21 wild-type retinas (Fig. 4A), as previously demonstrated (Pearson et al., 2010), as well as in the periphery of *Pten* cKOs (Fig. 4B). In contrast, ZO-1 staining was nearly absent in the P21 *Pten* cKO hamartoma-like region (Fig. 4B′). To confirm that the OLM was disrupted, we also examined the expression of N-cadherin (N-cad), which is an integral component of the cadherin-catenin complexes that make up the adherens junctions in the OLM. Similar to ZO-1, N-cad labelled a continuous line on top of the ONL in P21 wild-type retinas and in the *Pten* cKO periphery (Fig. 4C,D). In contrast, N-cad labelling was disrupted by gaps in the P21 *Pten* cKO hamartoma-like lesions (Fig. 4D′). Taken together, these observations suggest that the OLM is perturbed in the central hamartoma mass.

### The RPE is unaffected in *Pten* cKO hamartomas

We next examined mechanical integrity of the RPE, which is a layer of pigmented cells that also provides structural support on the apical side. To determine whether the RPE was intact in *Pten* cKOs, we stained P21 retinal sections with Otx2, which marks a subset of RPE cells as well as bipolar cells in the upper INL in wild-type retinas (Fig. 4E). In P21 *Pten* cKO retinas in the periphery, Otx2 expression was maintained in the RPE tissue overlying the retina, as well as in bipolar cells (Fig. 4F). However, within the hamartoma region in *Pten* cKO retinas, we were not able to determine whether the Otx2^+^ cells that were present were bipolar cells or RPE cells due to the large-scale disruption of tissue morphology (Fig. 4F′). We thus also examined the expression of RPE65, which is an enzyme expressed in the RPE that converts retinyl esters to 11-cis retinol (Sheridan et al., 2017). RPE65 expression was detected in a punctate fashion in the RPE of P21 wild-type (Fig. 4G) and *Pten* cKO (Fig. 4H,H′) retinas, including in the cell layer overlying the hamartoma and in the retinal rosettes within this structure. The RPE is thus intact in the periphery of *Pten* cKO retinas and is also present in the central hamartoma lesion.

### Disruption of the ILM on the basal side of *Pten* cKO retinas

Finally, we examined the ILM, which is a basement membrane consisting of laminin, collagen and fibronectin that contacts Müller glia endfeet to provide structural support on the basal side of the retina. In P21 wild-type retinas, laminin (Fig. 4I) and fibronectin (Fig. 4K) marked a thin, homogenous basal lamina. The basal lamina also appeared intact in the P21 *Pten* cKO peripheral retina, with laminin (Fig. 4J) and fibronectin (Fig. 4L) marking a continuous layer in the mutant tissue in the periphery. These markers were also clearly expressed within the P21 *Pten* cKO central hamartoma tissue, although there were more gaps in laminin (Fig. 4J′) and fibronectin (Fig. 4L′) expression, suggesting that the ONL may be thinner and less continuous in these lesions.

To further assess possible defects in the ILM in *Pten* cKO hamartomas, we performed electron microscopy. At the ultrastructural level, the ILM is divided into three layers: the lamina rara interna, an internal lamina densa and the lamina rara externa, which were all visible in P21 wild-type retinas (Fig. 4M,M′). The lamina rara externa makes contact with the vitreous, where collagen fibres are normally observed to protrude from the ILM (Fig. 4M,M′). A similar ultrastructural appearance was seen in P21 *Pten* cKO retinas in the periphery (Fig. 4N,N′), where *Pten* is deleted, suggesting that the ILM is mostly intact in the absence of *Pten.* In contrast, in the central ‘mosaically deleted’ hamartoma region in *Pten* cKO retinas, the ILM was thicker and appeared less organized, with additional fibrillary structures observed in the vitreous, suggestive of a disorganization of the ILM (Fig. 4O,O′).

Thus, in summary, the mosaic of mostly wild-type and some *Pten* cKO cells in the central retina results in structural defects in the OLM and, to a lesser extent, the ILM, which suggests that mechanical issues may contribute to hamartoma formation.

### Rapamycin limits hamartoma formation when administered in the early postnatal period, but it also induces new peripheral dysmorphologies in *Pten* cKO retinas

**Fig. 5.**
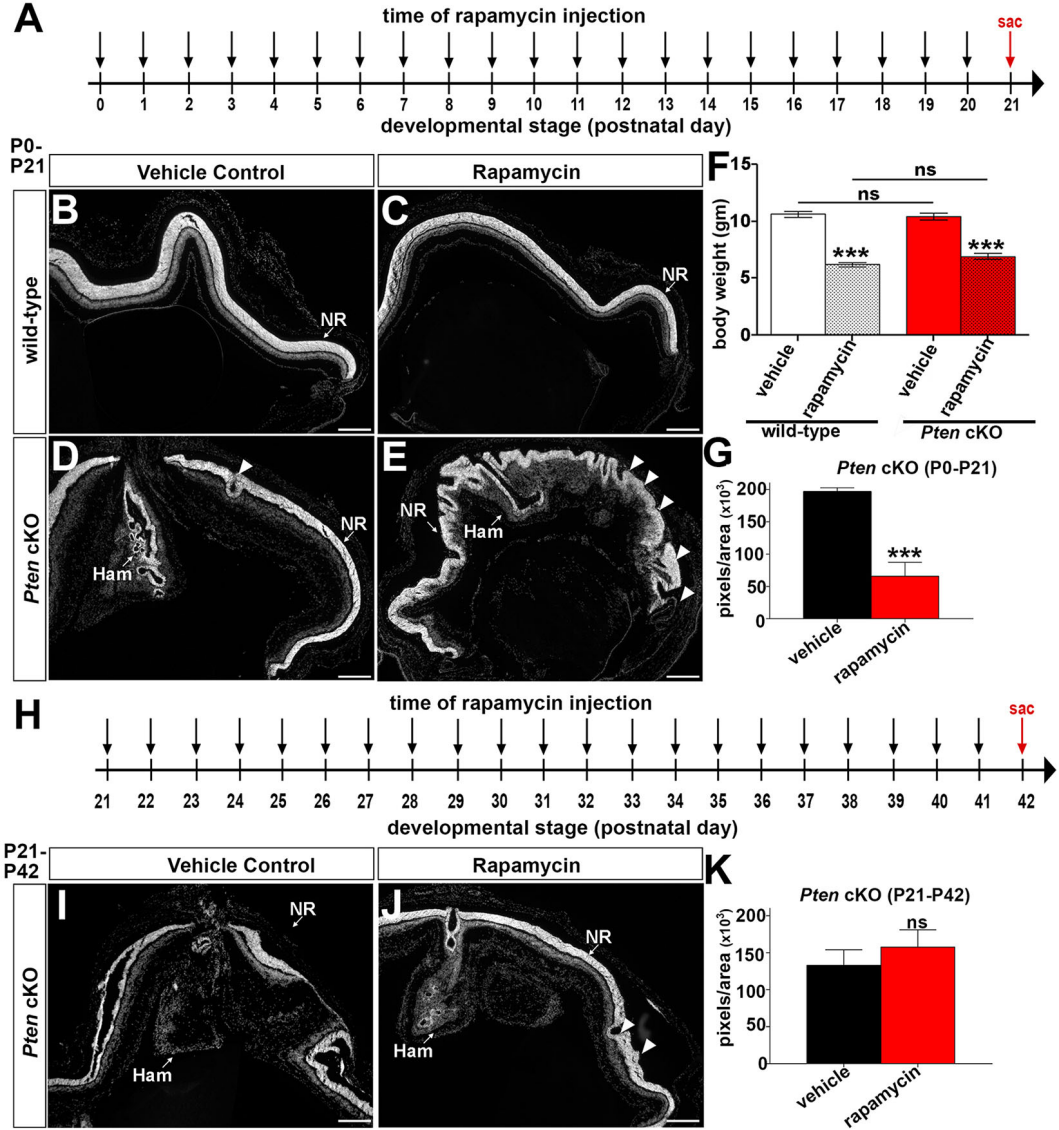
**Rapamycin reduces hamartoma size but also induces a new dysmorphology of the peripheral retina when administered in the early postnatal period in *Pten* cKOs.** (A) Experimental timeline for rapamycin and vehicle injection (P0-P21). Black arrows indicate the time of rapamycin or vehicle administration, and a red arrow indicates the time point when mice were collected (sac, sacrifice at P21). (B-E) DAPI images of P21 wild-type (B,C) and *Pten* cKO (D,E) retinas collected after vehicle control (B,D) or rapamycin (C,E) injections. Arrowheads in E mark the dysmorphology observed in *Pten* cKO retinas after rapamycin treatment. (F) Body weight measurements of wild-type and *Pten* cKO animals at P21, after 21 days of vehicle or rapamycin injections. (G) Measurement of hamartoma size in *Pten* cKO retinas after vehicle control or rapamycin injections for 21 days (P0-P21). (Wild-type control: *n*=7, *Pten* cKO control: *n*=5, wild-type rapamycin: *n*=8, *Pten* cKO rapamycin: *n*=5.) (H) Experimental timeline for rapamycin or vehicle injection (P21-P42). Black arrows indicate the time of rapamycin or vehicle administration, and red arrow indicates the time point when mice were collected (sac, sacrifice at P42). (I,J) DAPI images of P42 *Pten* cKO retinas collected after vehicle control (I) or rapamycin (J) injections beginning from P21. Arrowheads in J mark a few sites of dysmorphology observed in *Pten* cKO retinas after rapamycin treatment. (K) Measurement of hamartoma size in *Pten* cKO retinas after vehicle control or rapamycin injections for 21 days (P21-P42). (Wild-type control: *n*=9, *Pten* cKO control: *n*=9, wild-type rapamycin: *n*=9, *Pten* cKO rapamycin: *n*=9.) Ham, hamartoma; NR, neural retina. Scale bars: 200μm.

The main advantage of generating an animal model of PHTS is that it can be used to test the efficacy of drug treatments. Pten is a negative regulator of PI3K, and mTOR signalling is activated downstream of PI3K so, in the absence of PTEN, mTOR signalling is elevated (O'Reilly et al., 2006). An open-label drug trial has been started with the mTOR inhibitor sirolimus (alternative name for rapamycin) to treat PHTS patients, and this drug has shown some efficacy in inhibiting the growth of lipomas, which are a type of hamartoma (Leipert et al., 2016; Schmid et al., 2014; Wang et al., 2007). We previously reported that mTOR signalling is upregulated in *Pten* cKO retinas (Cantrup et al., 2012). We therefore asked whether treatment of *Pten* cKO retinas prior to hamartoma formation could prevent their subsequent development. To do so, wild-type and *Pten* cKO mice were administered rapamycin (2 mg/kg body weight) or vehicle control intraperitoneally (i.p.) daily from P0 to P21 (Fig. 5A). The animals survived rapamycin treatment but had 1.75-fold (wild-type; vehicle: 10.64±0.2644 g, rapamycin: 6.225±0.1830 g, *P*<0.0001) and 1.53-fold (*Pten* cKO; vehicle: 10.46±0.2960 g, rapamycin: 6.950±0.2500 g, *P*<0.0001) decreases in body weight (Fig. 5F), suggesting that, at this dosage, there are adverse effects on postnatal development and body size, as previously demonstrated (Fingar et al., 2002; Leontieva et al., 2012), precluding us from testing even higher doses.

To specifically assess the effects of rapamycin on the wild-type and *Pten* cKO retinas, we first examined the morphology of the treated eyes after daily injections between P0 and P21. In P21 wild-type retinas treated daily from P0 with rapamycin, the normal retinal morphology observed in vehicle controls was observed (Fig. 5B,C). In addition, all of the retinal cell types were present in the rapamycin-treated wild-type retinas, as revealed by the expression of rhodopsin (rods), cone arrestin (cones), calbindin (horizontal cells), Chx10 (bipolar cells), Sox9 (Müller glia), Pax6 (amacrine cells and RGCs) and Brn3b (RGCs) (Fig. S4). In contrast, in *Pten* cKOs, rapamycin treatment from P0 to P21 induced a striking ‘wavy’ dysmorphology of the peripheral retina, especially in the ONL, abnormalities that were not observed except in a few small areas in vehicle-treated *Pten* cKOs (Fig. 5D,E). Moreover, although all seven retinal cell types were present in the peripheral and central retina of rapamycin-treated *Pten* cKO animals, rosetting of the ONL disrupted the positioning of photoreceptors and underlying INL and GCL cells (Fig. S4). More importantly, within the central retina, rapamycin treatment successfully reduced hamartoma size compared to vehicle treatments in *Pten* cKOs (control: 196±21 pixels; rapamycin: 65±5 pixels, *P*<0.0001; Fig. 5G).

**Fig. 6.**
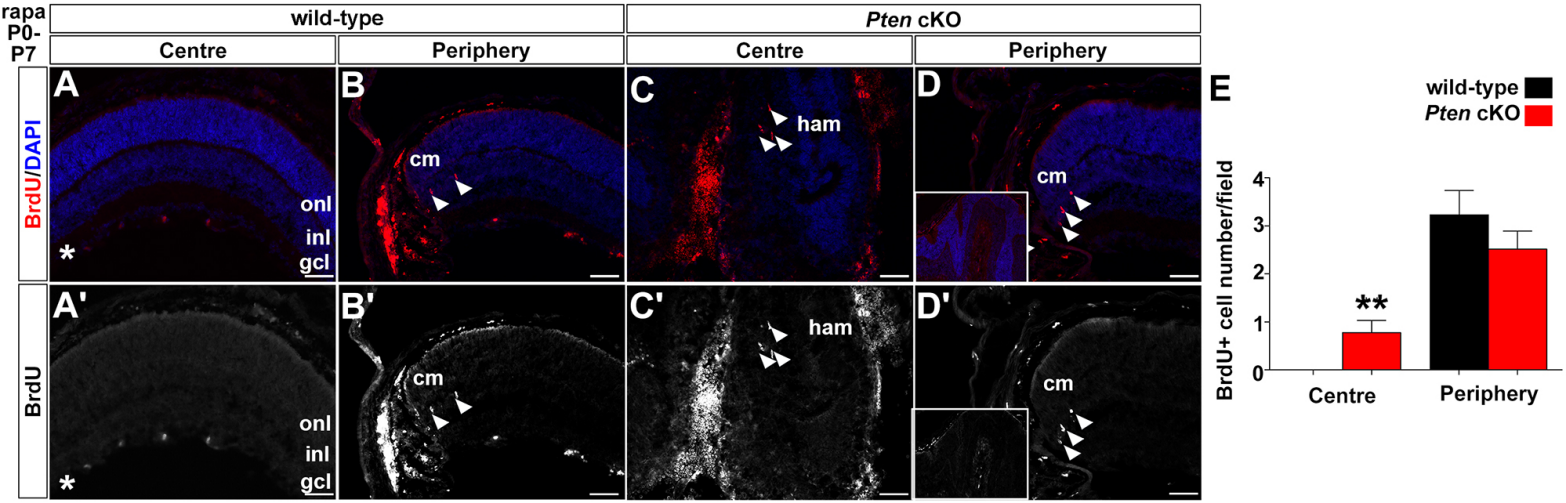
**Rapamycin** **treatment prior to the onset of hamartoma formation has only a very minor effect on proliferation in *Pten* cKO retinas.** (A-D′) Incorporation of BrdU in retinas from animals treated with rapamycin from P0-P7, including wild-type retinas in the centre (A,A′) and periphery (B,B′), and *Pten* cKO retinas in the central hamartoma (C,C′) and periphery (D,D’). Arrowheads mark BrdU^+^ cells. Blue is DAPI counterstain. (A′-D′) Desaturated red channel only. Asterisks indicate non-specific staining in blood vessels. (E) Quantitation of BrdU^+^ cells in the centre and periphery of wild-type (black bar) and *Pten* cKO (blue bar) retinas (wild-type rapamycin: *n*=6, *Pten* cKO rapamycin: *n*=8). gcl, ganglion cell layer; ham, hamartoma; inl, inner nuclear layer; onl, outer nuclear layer. Scale bars: 50 μm.

Rapamycin thus not only reduces hamartoma size but also has striking effects on retinal morphogenesis when administered in the early postnatal period, but only in the context of a *Pten* mutation. To determine whether these alterations in retinal morphology were due to change in cell proliferation, we injected rapamycin from P0 to P7, and then examined BrdU incorporation (after a 30 min injection; Fig. 6A-E), which labels S-phase cells. Rapamycin treatment from P0 to P7 did not increase the number of dividing cells in the central or peripheral retina compared to vehicle controls, with only a few ectopically dividing cells observed in the central retinal of *Pten* cKOs after rapamycin treatment (control: 0, rapamycin: 0.77±0.24, *P*<0.003), but a few proliferating cells were also observed in control animals (Fig. 6E,J versus Fig. S2E). Notably, rapamycin treatment between P0 and P21 in wild-type (Fig. S3B,B′,F,F′) or *Pten* cKO (Fig. S3D,D′,H,H′) retinas also did not induce ectopic cell proliferation as assessed by Ki67 expression. We thus can conclude that rapamycin is not inducing ectopic cell division in the *Pten* cKO retina, suggesting again that structural defects are likely the underlying cause of the dysmorphology.

### Rapamycin treatment decreases mTOR and Akt signalling in the retina

**Fig. 7.**
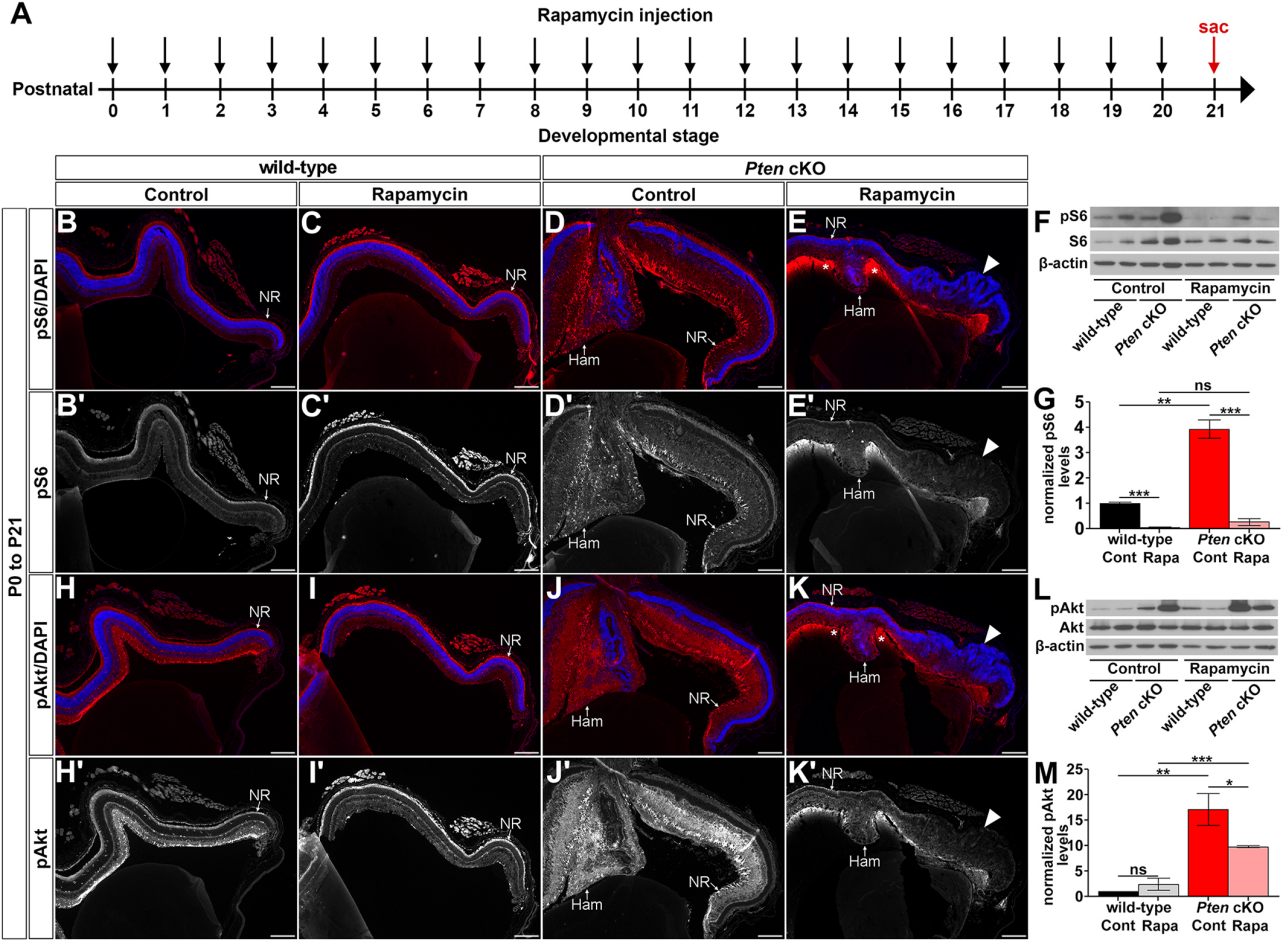
**Rapamycin treatment beginning at P0, prior to the onset of hamartoma formation, worsens defects in the retinal architecture in *Pten* cKO retinas.** (A) Schematic of experimental timeline. Black arrows indicate rapamycin administration, and red arrow indicates the time point when mice were collected (sac, sacrifice at P21). (B-E′) P21 wild-type and *Pten* cKO retinas collected from animals administered vehicle alone (B,B′,D,D′) or rapamycin (C,C′,E,E′) for 21 days (from P0) and immunolabelled with pS6^Ser235/236^ (wild-type control: *n*=7, *Pten* cKO control: *n*=5, wild-type rapamycin: *n*=8, *Pten* cKO rapamycin: *n*=5). (F,G) Western blot (F) and densitometry (G) of pS6^Ser235/236^ labelling of P21 wild-type and *Pten* cKO retinal lysates treated with vehicle alone or rapamycin (wild-type control: *n*=9, *Pten* cKO control: *n*=9, wild-type rapamycin: *n*=9, *Pten* cKO rapamycin: *n*=9). (H-K′) P21 wild-type and *Pten* cKO retinas collected from animals administered vehicle alone (H,H′,J,J′) or rapamycin (I,I′,K,K′) for 21 days (from P0) and immunolabelled with pAkt^Ser473^ (wild-type control: *n*=7, *Pten* cKO control: *n*=5, wild-type rapamycin: *n*=8, *Pten* cKO rapamycin: *n*=5). (L,M) Western blot (L) and densitometry (M) of pAkt^Ser473^ labelling of P21 wild-type and *Pten* cKO retinal lysates treated with vehicle alone or rapamycin (wild-type control: *n*=9, *Pten* cKO control: *n*=9 from three litters; wild-type rapamycin: *n*=9, *Pten* cKO rapamycin: *n*=9). Blue is a DAPI counterstain. Arrowheads mark the dysmorphic regions of the retina in *Pten* cKOs treated with rapamycin (E,E′,K,K′). Ham, hamartoma; NR, neural retina. Scale bars: 200 μm. *P<0.05; **P<0.01; ***P<0.001.

To further characterize the effects of rapamycin administered from P0 to P21 (Fig. 7A), we stained retinal sections for phosphorylated S6 (pS6), which is a part of the 40S ribosomal subunit that is a phosphorylation target for 70S6 kinase, and which is activated and phosphorylated by mTOR (Magnuson et al., 2012). In the P21 wild-type eye, very low, diffuse pS6 expression was detected (Fig. 7B,B′), which is consistent with previous reports in the mature eye (Sheridan et al., 2017). Levels of pS6 appeared even lower in P21 wild-type retinas treated with rapamycin for 21 days (Fig. 7C,C′), which we confirmed by western blotting (*n*=4; control: 1.00±0.06 normalized pS6 protein levels, rapamycin: 0.06±0.02 normalized pS6 protein levels, *P*<0.001; Fig. 7F,G). In P21 untreated *Pten* cKO retinas, pS6 expression was elevated in both peripheral and hamartoma regions of the retina compared to wild-type controls (Fig. 7D,D′), consistent with our previous study (Cantrup et al., 2012) and confirmed by western blotting (*n*=4; 3.94±0.37 normalized pS6 protein levels, *P*<0.01; Fig. 7F,G). In addition, treatment of *Pten* cKO retinas with rapamycin for 21 days drastically reduced the levels of pS6 detected in *Pten* cKO retinas, even lower than the pS6 levels in untreated wild-type retinas (*n*=4; 0.27±0.13 normalized pS6 protein levels, *P*<0.005; Fig. 7E,E′,F,G). Rapamycin therefore had the desired effect in reducing mTOR signalling.

It was previously shown that rapamycin upregulates Akt phosphorylation (Leipert et al., 2016; O'Reilly et al., 2006; Schmid et al., 2014), which is already elevated in *Pten* cKO retinas (Cantrup et al., 2012; Tachibana et al., 2016). We thus asked whether pAkt levels were further increased in animals treated with rapamycin. pAkt labels cells in the inner retina in P21 wild-type retinas, and the levels were not altered by the treatment with rapamycin (*n*=4; control: 1.00±0.03 normalized Akt protein levels; rapamycin: 2.41±1.19, *P*=0.36; Fig. 7H-K″,L,M). However, in P21 *Pten* cKO retinas, pAkt levels were greatly increased compared to wild-type (*n*=4; 17.13±3.16 normalized Akt protein levels, *P*=0.007; Fig. 7J,J″,L,M). Interestingly, contradicting previous studies, rapamycin reduced pAkt levels in *Pten* cKO retinas, but not to wild-type levels, such that pAkt signalling remained elevated (*n*=4; 9.80±0.18 normalized Akt protein levels, *P*=0.040; Fig. 7K-K″,L,M).

### Treatment with rapamycin after P21 does not limit hamartoma size or grossly alter retinal morphology

We next questioned whether rapamycin could limit hamartoma size in *Pten* cKO retinas when administered after hamartoma formation. To do so, rapamycin was injected daily starting at P21, when hamartoma formation was fully penetrant, and continuing for 21 days until P42, treating both wild-type and *Pten* cKO mice (Fig. 5H). Strikingly, rapamycin treatment from P21 to P42 did not reduce hamartoma size in *Pten* cKOs, nor did it affect retinal morphology in wild-type or *Pten* cKOs (wild-type: 133±20 pixels; *Pten* cKO: 157±23 pixels, *P*=0.63; Fig. 5I-K). Furthermore, all retinal cell types were present in *Pten* cKO retinas after 21 days of rapamycin treatment, both in the central hamartoma and the peripheral retina (Fig. S5).

**Fig. 8.**
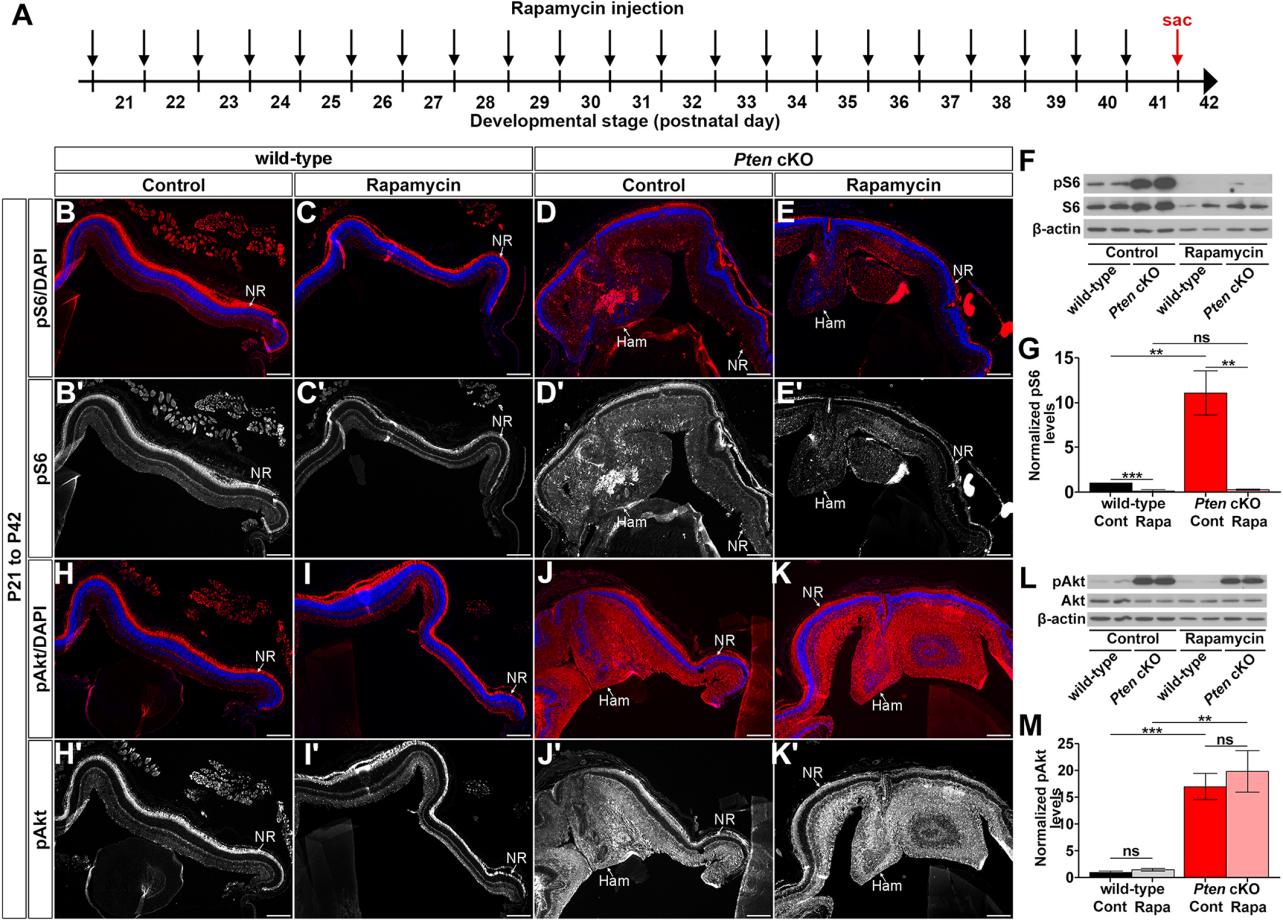
**Rapamycin treatment does not reduce hamartoma size in *Pten* cKOs when administered after P21.** (A) Schematic of experimental timeline. Black arrows indicate rapamycin administration, and red arrow indicates the time point when mice were collected (sac, sacrifice at P42). (B-E′) P42 wild-type and *Pten* cKO retinas collected from animals administered vehicle alone (B,B′,D,D′) or rapamycin (C,C′,E,E′) for 21 days (from P21) and immunolabelled with pS6^Ser235/236^ (wild-type control: *n*=6, *Pten* cKO control: *n*=5, wild-type rapamycin: *n*=7, *Pten* cKO rapamycin: *n*=5). (F,G) Western blot (F) and densitometry (G) of pS6^Ser235/236^ labelling of P42 wild-type and *Pten* cKO retinal lysates treated with vehicle alone or rapamycin (wild-type control: *n*=9, *Pten* cKO control: *n*=9, wild-type rapamycin: *n*=9, *Pten* cKO rapamycin: *n*=9). (H-K′) P42 wild-type and *Pten* cKO retinas collected from animals administered vehicle alone (H,H′,J,J′) or rapamycin (I,I′,K,K′) for 21 days (from P21) and immunolabelled with pAkt^Ser473^ (wild-type control: *n*=6, *Pten* cKO control: *n*=5, wild-type rapamycin: *n*=7, *Pten* cKO rapamycin: *n*=5). (L,M) Western blot (L) and densitometry (M) of pAkt^Ser473^ labelling of P42 wild-type and *Pten* cKO retinal lysates treated with vehicle alone or rapamycin (wild-type control: *n*=9, *Pten* cKO control: *n*=9, wild-type rapamycin: *n*=9, *Pten* cKO rapamycin: *n*=9). Blue is a DAPI counterstain. Arrowheads mark the dysmorphic regions of the retina in *Pten* cKOs treated with rapamycin (E,E′,K,K′). Ham, hamartoma; NR, neural retina. Scale bars: 200 μm. **P<0.01; ***P<0.001.

We ensured that rapamycin had the desired effects when administered between P21 to P42 (Fig. 8A) by immunolabelling retinal sections for pS6. Continuous treatment with rapamycin from P21 to P42 resulted in a significant reduction in pS6 levels in both wild-type and *Pten* cKO retinas compared to vehicle controls (*n*=4; wild-type control: 1.00±0.039 normalized pS6 protein levels, wild-type rapamycin: 0.18±0.098, *P*=0.0002; *Pten* cKO control: 11.13±2.47 normalized pS6 protein levels, *Pten* cKO rapamycin: 0.32±0.10 normalized pS6 protein levels, *P*=0.0047; Fig. 8B-E′,F,G), as shown with the rapamycin treatment at the earlier postnatal stage (i.e. from P0 to P21; Fig. 7B-E′,F,G). We also analyzed the effect of rapamycin treatment from P21 to P42 on Akt phosphorylation. Surprisingly, no significant alterations in Akt phosphorylation were observed in both wild-type and *Pten* cKO retinas after 21 days of rapamycin treatment (P21-P42) compared to controls (*n*=4; wild-type control: 1.00±0.30 normalized p-AKT protein levels, wild-type rapamycin: 1.47±0.26 normalized p-AKT protein levels, *P*=0.28; *Pten* cKO control: 17.08±2.48 normalized p-AKT protein levels, *Pten* cKO rapamycin: 19.88±3.87 normalized p-AKT protein levels, *P*=0.56; Fig. 8H-K′,L,M). Maintenance of high pAkt expression in both treated and untreated *Pten* cKO retinas indicates that rapamycin cannot diminish the elevated Akt phosphorylation levels that arise due to *Pten* loss in mature retinas.

Taken together, administration of rapamycin after hamartoma formation has initiated fails to reduce the size of these lesions and, hence, rapamycin may not be an effective treatment strategy for PHTS patients with retinal and possibly other CNS lesions.

## DISCUSSION

Although PHTS is a rare disorder, the overall individual, familial and societal burden is significant as these are chronic, multi-systemic disorders starting from early childhood. Current research has almost exclusively focused on *PTEN* in malignant transformation, since somatic *PTEN* mutations are acquired in many aggressive cancers. While PHTS patients are predisposed to malignancy, many indicate that their greatest suffering comes from their so-called benign disease: soft tissue hamartomas that start growing in early childhood and lead to disfigurement and painful debilitation. Animal models have been used to elucidate *Pten* functions in developing neuronal (Backman et al., 2001; Cantrup et al., 2012; Groszer et al., 2001; Kwon et al., 2006; Tachibana et al., 2016), skeletal (Hsieh et al., 2009) and vascular (Hamada et al., 2005) lineages, amongst others, as well as to determine the role of *PTEN* as a tumour suppressor (Carnero and Paramio, 2014). However, efforts to model hamartomas have largely been unsuccessful, in part due to the early lethality of homozygous germline mutations in *Pten* (Di Cristofano et al., 1998) and other hamartoma-associated genes, such as tuberous sclerosis genes (*Tsc1/Tsc2*) (Kobayashi et al., 1999, 2001). Herein, we reported the generation of an animal model of PHTS in the retina, characterizing the underlying defects and testing the responsiveness of these lesions to rapamycin as a therapeutic strategy.

### *Pten* cKO retinas as a model of PHTS

It is striking that the hamartoma-like lesions observed in *Pten* cKOs form in the central retina, which is a mosaic of mostly ‘wild-type’ and some *Pten*-null cells. The reproducibility of these lesions is also surprising, especially given that, in PHTS patients, hamartomas demonstrate striking phenotypic heterogeneity and can develop in many different locations and involve multiple tissue components. How this heterogeneity arises is unknown, but a leading hypothesis is that second-hit mutations create cohorts of mutant cells that act non-cell autonomously to create disorder in neighbouring ‘normal’ tissue. Thus, the mosaic scenario we observe in *Pten* cKO retinas mimics to a certain extent what is going on in PHTS patients, who have germline loss-of-function mutations/deletions of one *PTEN* allele in all cells of the body, and possibly additional second-hit genetic modifications at sites of hamartoma formation. Indeed, two patients have been identified with biallelic inactivation of *PTEN*, either by compound-heterozygous mutations or by loss of heterozygosity (LOH) (Caux et al., 2007; Zhou et al., 2000). Both patients had extensive hamartoma involvement and were initially classified as Proteus-like syndromes.

It is not known whether similar mechanisms underlie the more typical hamartomas seen in PHTS with germline mutations in one *PTEN* allele. Additional support for a LOH mechanism is found in the tuberous sclerosis complex hamartoma disorders caused by mutations in *TSC1/TSC2* (Giannikou et al., 2016; van Eeghen et al., 2012). Alternatively, hamartomas could result from permissive alleles introduced by mutations in related genes, such as concomitant *SDHB-D* mutations or *KLLN* promoter hypermethylation, which are thought to confer a greater risk of malignant transformation (Bennett et al., 2010; Mahdi et al., 2014; Ni et al., 2012), or in another component of the PI3K-AKT signalling pathway. Finally, it is not known whether these second hits occur as regional events in all cell types within a hamartoma or, more likely, as intra-lesional somatic mosaicism in a specific type(s) of cell(s) that then influences its neighbours in a non-cell-autonomous manner.

Support for a non-cell-autonomous mechanism underlying hamartoma formation comes from the demonstration that CLOVES syndrome patients, who also develop hamartomas, have activating mutations in *PIK3CA*, which phenocopies *PTEN* loss (Kurek et al., 2012b). Strikingly, *PIK3CA* activating mutations occur in as few as 3-30% of cells within the dysmorphic tissue, suggesting that mutant cells can influence the organization of surrounding wild-type tissue (Kurek et al., 2012b; Luks et al., 2015). Our retinal model of PHTS further supports a non-cell-autonomous model for hamartoma formation, as the hamartoma-like lesions developed in a ‘mosaic’ of wild-type and *Pten* mutant cells in the central retina, and not in the *Pten* cKO periphery, where gene deletion was essentially complete. Currently, it is not known how *Pten* mutant cells communicate with neighbouring wild-type cells to create disorder.

### Structural defects underlie hamartoma formation in the *Pten* cKO retina

Our data suggests that hamartoma formation may arise in part due to mechanical deficits in the retina. Indeed, we identified several structural defects in the *Pten* cKO hamartoma, including disruption of the OLM and ILM. Previous studies have shown that pharmacological disruption of the OLM with aminoadipic acid also perturbs retinal morphology, resulting in a transient rosetting of the ONL that later resolves as the OLM is repaired and the drug washed out of the system (Pearson et al., 2010). In addition, disruption of the ILM, which provides structural support to softer neural cells (Galli-Resta et al., 2008), results in retinal ectopias in several diseases [e.g. Walker–Warburg syndrome (Galli-Resta et al., 2008)], and the ILM is also critical to maintain intraocular pressure and control tissue growth (Halfter et al., 2006). An integral component of the ILM and OLM are the Müller glia, and the positioning and morphology of these cells was also disrupted in *Pten* cKO mutant and ‘wild-type’ hamartoma tissue. Müller glia are critical for retinal cell positioning, as conditioned media from Müller glia can re-introduce order in disaggregated retinal cells (Layer et al., 2002). It is thus likely that the combined disruption of Müller glia, the ILM and OLM contributes to the formation of *Pten* cKO hamartomas in the central ‘mosaic’ region, altogether resulting in a loss of mechanical support that may result in unequal tissue forces inducing tissue malformations. Whether a similar disruption of tissue forces underlies hamartoma formation in other regions of the CNS, and in other tissues, remains to be determined.

### Hamartoma treatment with rapamycin

Current treatment for PHTS patients involves genetic counselling, surveillance for cancer, which these patients are prone to, and the alleviation of symptoms. In some instances, surgery can reduce hamartoma burden, but such an approach is challenging as the disease is multifocal and hamartomas can infiltrate other tissues (e.g. muscle). Nevertheless, because PHTS hamartomas comprise non-transformed cells, they may be highly amenable to correction using novel therapies targeting cell growth and patterning. The search for a pharmacological therapy requires the implementation of *in vitro* and, ideally, *in vivo* models for drug testing. One of the best *in vitro* models developed to date has been lipoma cell cultures derived from patients carrying germline mutations in *PTEN*, which are sensitive to sirolimus (the clinical name for rapamycin), which slows their growth (Leipert et al., 2016; Schmid et al., 2014; Wang et al., 2007).

Notably, the growth inhibitory effects of sirolimus against lipoma cells *in vitro* have not been matched *in vivo* (Schmid et al., 2014). The discrepancy between *in vitro* and *in vivo* data may be due in part to the inability of *in vitro* cell-based screens to recapitulate disease complexity, including cell-cell interactions. Isolated case reports have shown some benefit from AKT-mTOR pathway inhibition using sirolimus in open-label trials in PHTS patients (Iacobas et al., 2011; Marsh et al., 2008), whereas other reports have shown more limited efficacy (Schmid et al., 2014). The efficacy of sirolimus also plateaus after several months and is not durable following cessation (Marsh et al., 2008). It is also unclear how long-term suppression of this vital pathway will affect growth and development during childhood and adolescence, presumably the optimal window for treatment. Indeed, as seen in our study, treatment with rapamycin in the early postnatal period has devastating effects on retinal development.

We used our retinal model of PHTS to assess the efficacy of rapamycin and, although we did observe a reduction in hamartoma size, rapamycin also induced new retinal dysmorphologies in the peripheral retina that make it a poor candidate as a drug therapy on its own. A consideration for the future is that pAkt levels are elevated in *Pten* cKO retinal cells, and they remain elevated even after rapamycin treatment, so combining rapamycin with a drug targeting PI3K may be beneficial. Indeed, in lipoma cells, the growth-inhibitory effects of sirolimus are improved when combined with resveratrol, which targets p70S6K (Leipert et al., 2016; Schmid et al., 2014; Wang et al., 2007), suggesting that combined therapies may also improve the efficacy of rapamycin in treating retinal hamartomas.

In summary, with our animal model of PHTS in the retina, we can better characterize the disease mechanism and we may uncover new druggable targets for therapeutics that can arrest or minimize the PHTS disease burden. We may also generate new animal models of CNS hamartomas using the same premise, which is to create a scenario where wild-type and *Pten* mutant cells are intermingled, providing further support for the idea that hamartoma formation occurs in a mosaic of wild-type and *Pten* mutant cells.

## MATERIALS AND METHODS

### Animals

All animal procedures were approved by the Sunnybrook Research Institute Animal Care Committee, Canada in agreement with the Guidelines of the Canadian Council of Animal Care (CCAC). The *Pax6::Cre* driver (Marquardt et al., 2001) and *Pten*^fl^ allele (Backman et al., 2001) were generated previously and PCR genotyping was performed as described. Animals were maintained on a 129/Sv background. Sex was not considered and both male and female animals were grouped.

### Tissue processing and cryostat sectioning

Animals were sacrificed and their eyes were removed at the postnatal stages described in the text. Retinas were dissected out and examined macroscopically for hamartoma formation before being fixed overnight at 4°C in 4% paraformaldehyde (PFA) diluted in phosphate buffered saline (PBS), pH 7.5. The next day, retinas were washed 3×10 min in PBS, and then immersed in 20% sucrose/1× PBS overnight. Eyes were then embedded in optimal cutting temperature (OCT) compound on dry ice, and stored at −80°C before sectioning on a cryostat at 10 µm. Slides were stored at −20°C prior to immunohistochemistry.

### Immunohistochemistry

Cryosections were blocked in 10% horse serum, 0.1% Triton X-100 in PBS (PBT), pH 7.5, for 1 h before adding primary antibodies diluted in blocking solution. The following primary antibodies were used (anti-): pAkt^Ser473^ (1:500; Cell Signaling #4060), pS6^Ser235/236^ (1:500; Cell Signaling #4856), Brn3b (1:500; Chemicon #AB5945), Chx10 (1:200; Santa Cruz #sc-21690), cone-arrestin (1:500; Millipore #AB15282), Pax6 (1:500; Covance Research #PRB-278P), Pten (1:500; Cell Signaling #9559), rhodopsin (1:500; Chemicon #MAB5356), calbindin (1:500; Sigma #C9848), GFAP (1:500; Sigma #G9269), CRALBP (1:500; Abcam #ab15051), glutamine synthetase (1:500; Abcam #ab73593), fibronectin (1:200; Abcam #ab2413), laminin (1:500; Sigma #L9393), RPE65 (1:500; ORIGENE #TA309839), Otx2 (1:500; Abcam #ab21990), N-cadherin (1:200; BD Transduction Labs #610920), ZO-1 (1:100; ThermoFisher Scientific #33-9100) and Sox9 (1:500; Millipore #AB5535). Slides were incubated in primary antibodies overnight at 4°C. The next day, slides were washed three times in PBT before incubating with secondary antibodies conjugated with Alexa Fluor 568 (1:500; Molecular Probes) or Alexa Fluor 488 (1:500; Molecular Probes) for 1 h. Slides were then washed three times in PBT before labelling nuclei with DAPI.

### Western blotting

Retinas were collected from postnatal pups at the indicated stages, lysed in RIPA buffer with protease (1× protease inhibitor complete, 1 mM PMSF) and phosphatase (50 mM NaF, 1 mM NaOV) inhibitors, and 10 µg of lysate was run on SDS-PAGE gels for western blot analysis as described previously (Ma et al., 2007; Tachibana et al., 2016). Primary antibodies included (anti-): pAkt^Ser473^ (1:1000; Cell Signaling #4060), total Akt (1:1000; Cell Signaling #9272), pS6^Ser235/236^ (1:1000; Cell Signaling #4856), total S6 (1:1000; Cell Signaling #2217) and β-actin (1:10,000; Abcam #8227). Each western blot was performed three times on three sets of two independent samples, and densitometries were calculated using UN-SCAN-IT gel densitometry software (Silk Scientific). The average values of normalized expression levels were plotted.

### Drug administration

The mice were injected i.p. with 2 mg/kg body weight rapamycin (LC Laboratories, Woburn, MA) or vehicle (0.25% PEG, 0.25% Tween-80) beginning on P0 or P21, and injected daily for 7 or 21 days, as indicated for each experiment. This dose was selected as being within the lower range of 1.5 mg/kg to 10 mg/kg used in previous studies (Fang et al., 2013; Johnson et al., 2013; Kunchithapautham et al., 2011; Leontieva et al., 2014). Animals were sacrificed after 21 days of rapamycin treatment. The retinas were processed for immunohistochemistry and western blot as described above.

### Imaging, quantification and statistical analysis

Digital photomicrographs were captured under bright-field (DIC) or fluorescent light (fluorescence IHC) using a Leica DFC345FX camera attached to a Leica DMI8 microscope using Leica Application Suite X (LASX) imaging software (ver. 2.0.0.14332.2). All analyses were performed on a minimum of three eyes per genotype and a minimum of three photomicrographs per eye. Statistical significance was calculated using unpaired two-tailed Student's *t*-tests (to compare two samples) or one-way ANOVA (for multiple sample comparisons, with a Tukey *post hoc* analysis) using GraphPad Prism Software version 5.0 (GraphPad Software). Error bars represent standard error of the mean (s.e.m.).

## Supplementary Material

Supplementary information
