# Supplementary material for: Hamartoma-like lesions in the mouse retina: an animal model of Pten hamartoma tumour syndrome
Source: Dis Model Mech. 2018 May 21;11(5):dmm031005. doi: 10.1242/dmm.031005 (PMC5992614; doi:10.1242/dmm.031005)
Supplement: Supplementary information [file dmm-11-031005-s1.pdf]

SUPPLEMENTARY FIGURES

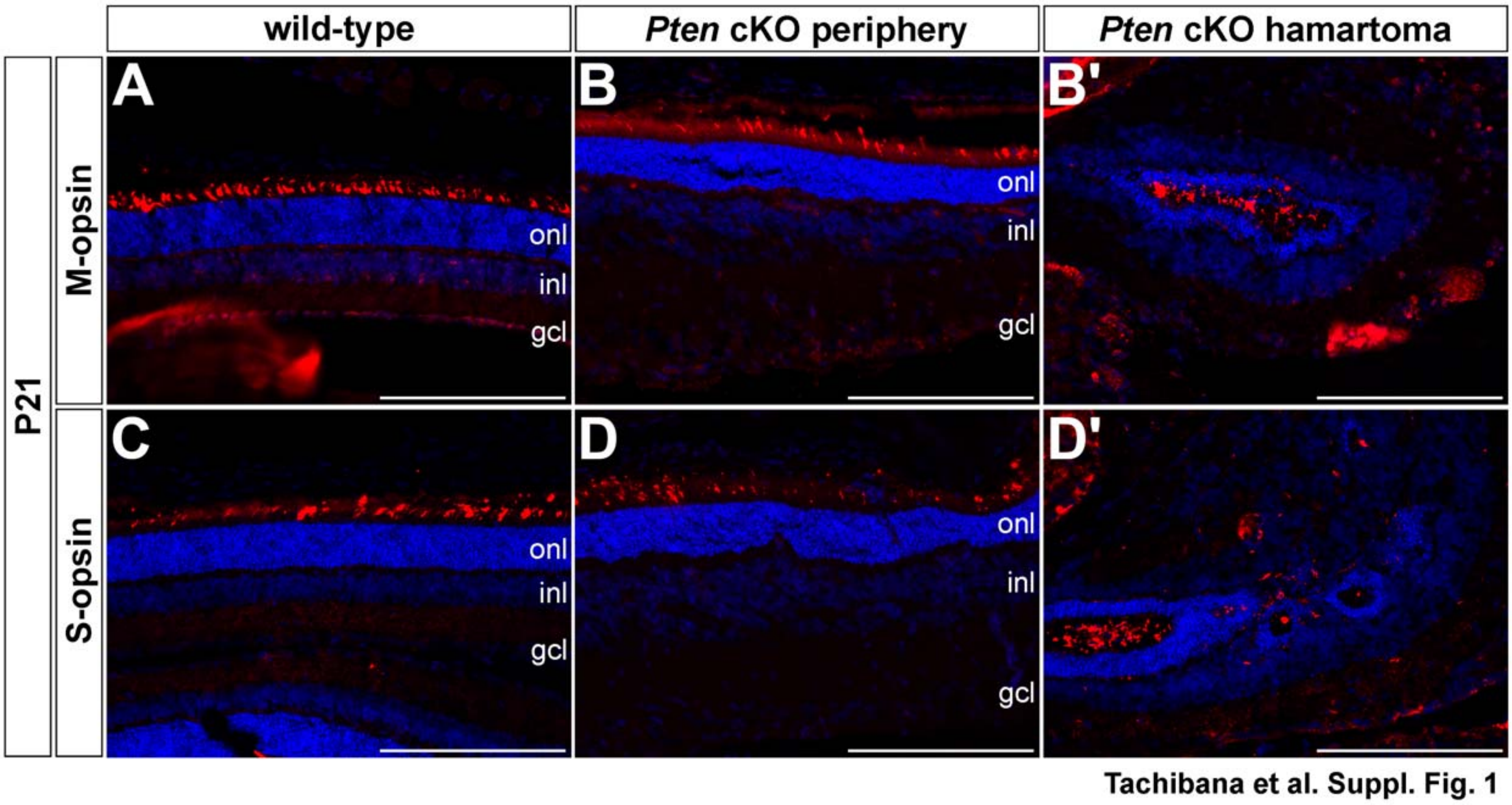

Supplementary Figure 1. Expression of M-opsin and S-opsin in P21 wild-type and *Pten* cKO retinas. (A-D') Immunolabeling of P21 wild-type and *Pten* cKO retinas for M-opsin (A-B'), S-opsin (C-D'). Blue is a DAPI counterstain. gcl, ganglion cell layer; Ham, hamartoma; inl, inner nuclear layer; NR, neural retina; onl, outer nuclear layer. Scale Bar: 200μm.

Disease Models & Mechanisms • Supplementary information

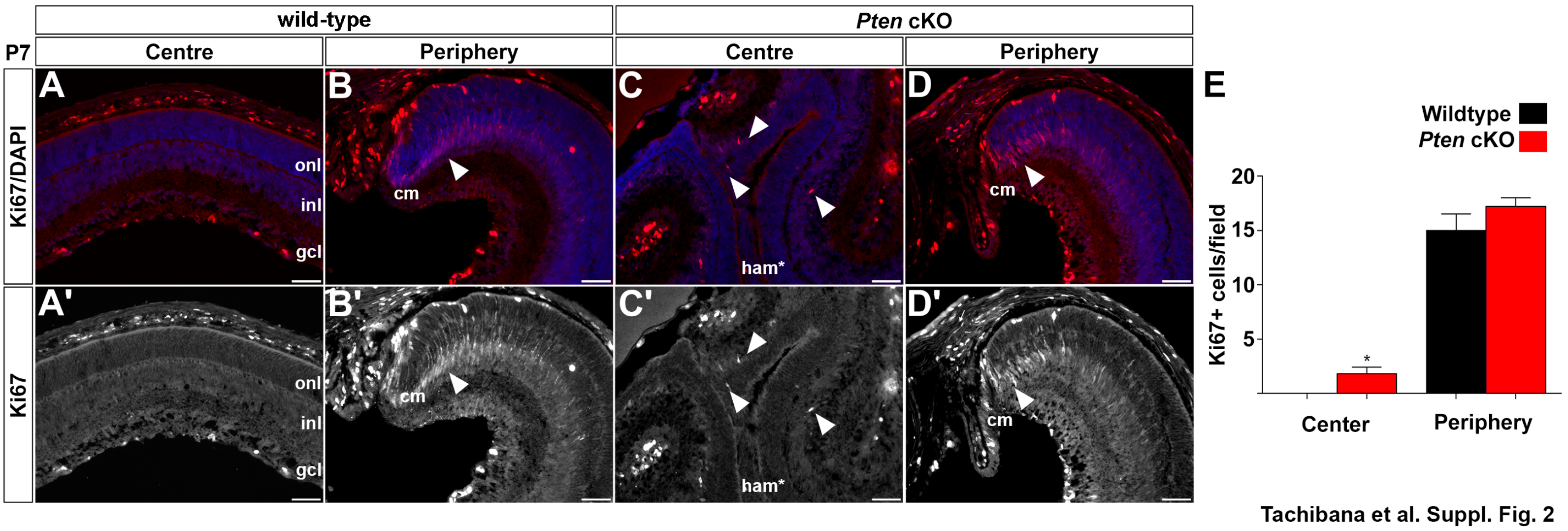

Supplementary Figure 2. Small increase in proliferation in the central retina in P7 *Pten* cKO retinas. (A-D) Expression of Ki67 in P7 wild-type (A,B,A',B') and *Pten* cKO (C,D,C',D') retinas. (wild-type: n=6, *Pten* cKO: n=6). Blue is a DAPI counterstain in A-D, and A'-D' are desaturated images of the red Ki67 channel. cm, ciliary margin; gcl, ganglion cell layer; ham, hamartoma; inl, inner nuclear layer; onl, outer nuclear layer. Scale Bar: 50 µm.

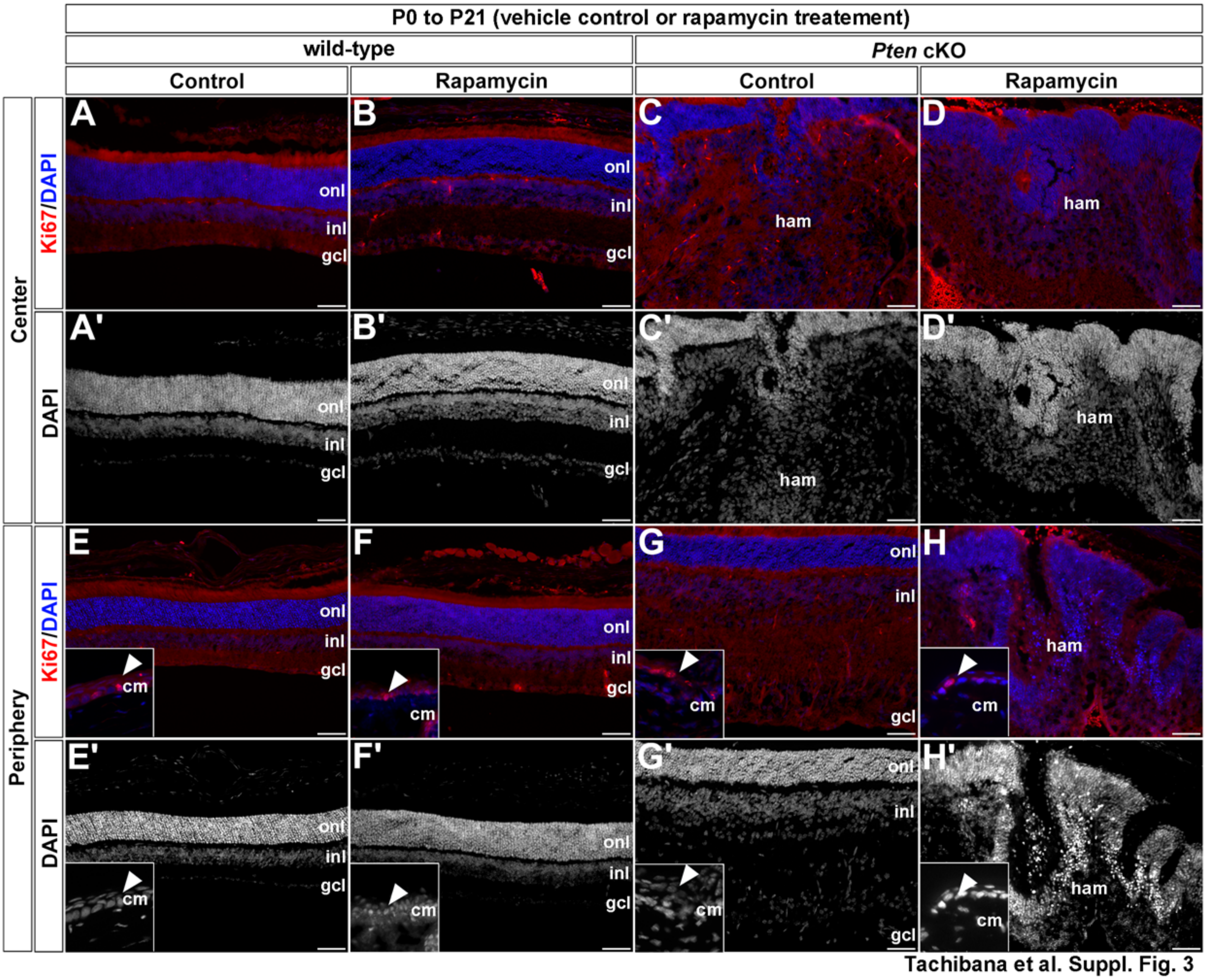

Supplementary Figure 3. Rapamycin has no effect on the proliferation of retinal cells in a P0 to P21 treatment regimen. (A-H) Expression of Ki67 in P21 wild-type retinas treated with vehicle (A,A',E,E') or rapamycin (B,B',F,F') from P0 to P21. (A-H) Expression of Ki67 in P21 *Pten* cKO retinas treated with vehicle control (C,C',G,G') or rapamycin (D,D',H,H') from P0 to P21. (wild-type control: n=7, *Pten* cKO control: n=5, wild-type rapamycin: n=8, *Pten* cKO rapamycin: n=5). Blue is a DAPI counterstain in A-H, and A'-H' are desaturated images of the red Ki67 channel. cm, ciliary margin; gcl, ganglion cell layer; ham, hamartoma; inl, inner nuclear layer; onl, outer nuclear layer. Scale Bar: 50 µm.

Disease Models & Mechanisms • Supplementary information

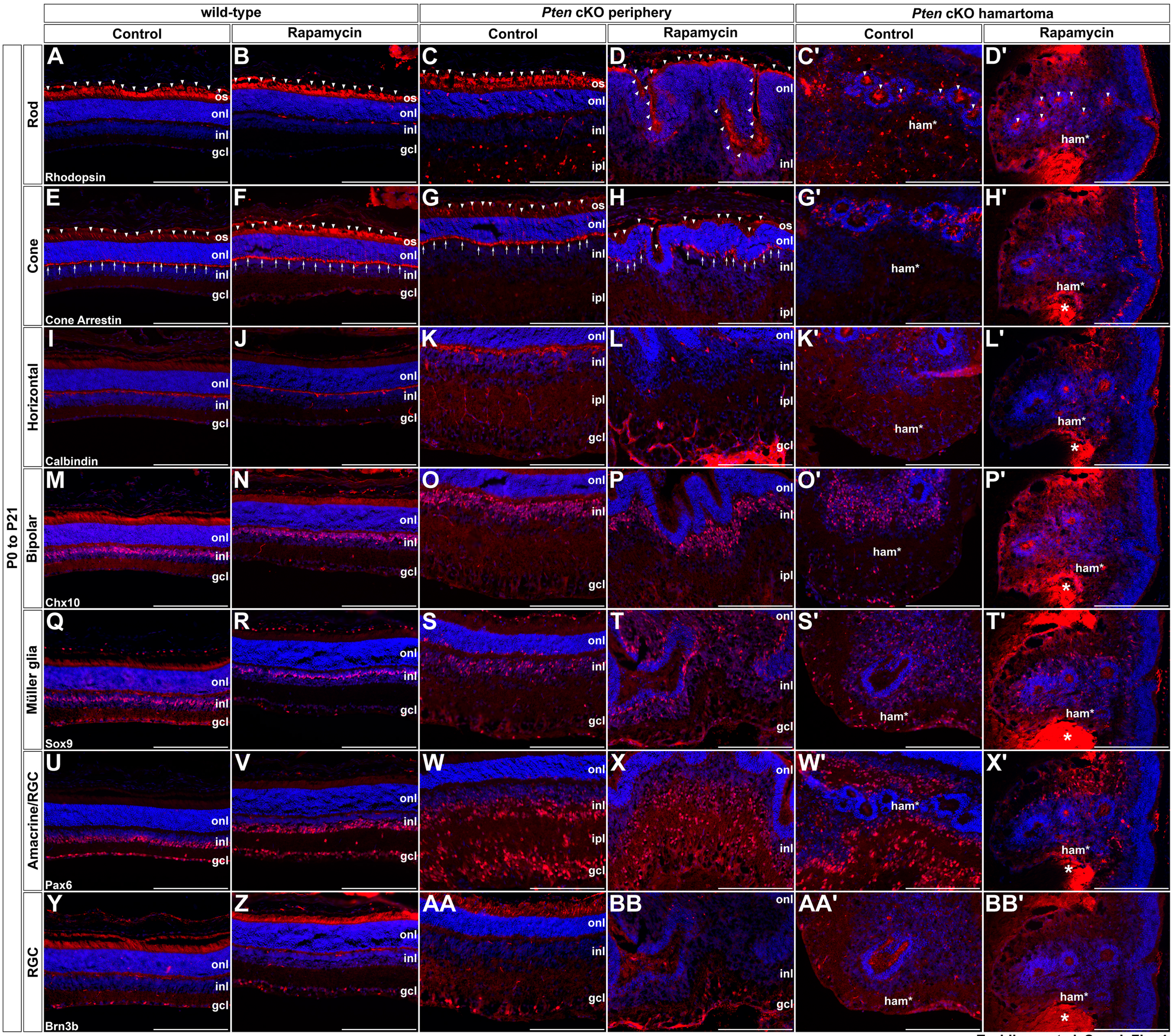

Tachibana et al. Suppl. Fig. 4

Supplementary Figure 4. Rapamycin treatment has no effect on the production of retinal cells when administered from P0-P21. (A-BB') Immunolabeling of vehicle control or rapamycin treated (P0-P21) wild-type and *Pten* cKO retinas for Rhodopsin (A-D,C'-D'), Cone Arrestin (E-H,G'-H'), Calbindin (I-L,K'-L'), Chx10 (M-P,O'-P'), Sox9 (Q-T,S'-T'), Pax6 (U-X,W'-X'), and Brn3b (Y-BB,AA'-BB'). Asterisks mark the autofluorescent blood outside retina in D',H',L',P',T',X',BB'. gcl, ganglion cell layer; inl, inner nuclear layer; onl, outer nuclear layer. Scale Bar: 200µm.

Disease Models & Mechanisms • Supplementary information

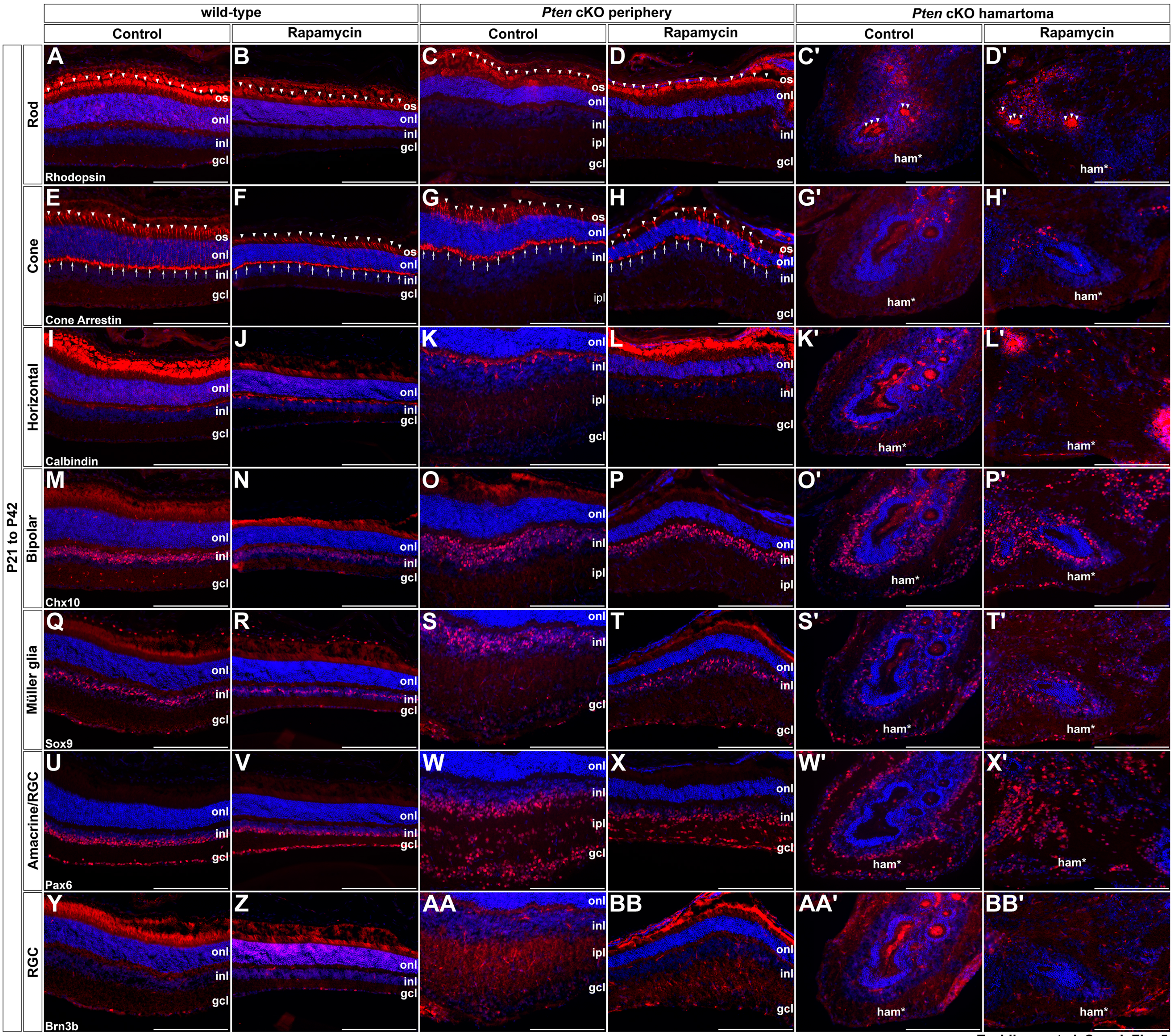

Supplementary Figure 5. Rapamycin treatment has no effect on the production of retinal cells when administered from P21 to P42. (A-BB') Immunolabeling of vehicle control or rapamycin treated wild-type and *Pten* cKO retinas at P42 for Rhodopsin (A-D,C'-D'), Cone Arrestin (E-H,G'-H'), Calbindin (I-L,K'-L'), Chx10 (M-P,O'-P'), Sox9 (Q-T,S'-T'), Pax6 (U-X,W'-X'), and Brn3b (Y-BB,AA'-BB'). gcl, ganglion cell layer; inl, inner nuclear layer; onl, outer nuclear layer. Scale Bar: 200µm.

Disease Models & Mechanisms • Supplementary information
